# The feasibility of equilibria in large ecosystems: A primary but neglected concept in the complexity-stability debate

**DOI:** 10.1371/journal.pcbi.1005988

**Published:** 2018-02-08

**Authors:** Michaël Dougoud, Laura Vinckenbosch, Rudolf P. Rohr, Louis-Félix Bersier, Christian Mazza

**Affiliations:** 1 Department of Mathematics, University of Fribourg, Fribourg, Switzerland; 2 University of Applied Sciences Western Switzerland - HES-SO, Yverdon-les-Bains, Switzerland; 3 Department of Biology, Unit of Ecology and Evolution, University of Fribourg, Fribourg, Switzerland; University of New South Wales, AUSTRALIA

## Abstract

The consensus that complexity begets stability in ecosystems was challenged in the seventies, a result recently extended to ecologically-inspired networks. The approaches assume the existence of a feasible equilibrium, i.e. with positive abundances. However, this key assumption has not been tested. We provide analytical results complemented by simulations which show that equilibrium feasibility vanishes in species rich systems. This result leaves us in the uncomfortable situation in which the existence of a feasible equilibrium assumed in local stability criteria is far from granted. We extend our analyses by changing interaction structure and intensity, and find that feasibility and stability is warranted irrespective of species richness with weak interactions. Interestingly, we find that the dynamical behaviour of ecologically inspired architectures is very different and richer than that of unstructured systems. Our results suggest that a general understanding of ecosystem dynamics requires focusing on the interplay between interaction strength and network architecture.

## Author summary

Finding conditions leading to stable equilibria, i.e. equilibria robust to small perturbations, is a key feature in understanding how persistent are ecological systems. The number of species involved in the ecosystem as well as the number of relations between them has historically been pointed out to be the prominent ingredients determining stability. The latter is however always computed for a particular equilibrium, which needs and is usually assumed to contain only positive abundances. This assumption has until now only barely been tested in relation to system size. We provide here analytical results complemented by numerical simulations which show that it is almost impossible to find equilibria containing only positive abundances in species rich systems or, if so, the parameters are constrained to lead to ecologically-nonsensical abundances in unstructured systems. Interestingly, only ecologically-inspired architectures permit one to obtain feasible equilibria with a rich and sensible dynamical behavior.

## Introduction

A central question in ecology is to understand the factors and conditions that ensure ecological systems to persist, a requisite for the sustained provisioning of vital ecosystem services. This question of a “balance of nature” has a long history in science [1, 2], and the consensus that “complexity begets stability” emerged among ecologists in the fifties. MacArthur [3] had a radical view on this question, arguing that stability will increase with the two fundamental ingredients of complexity, the number of species and of interactions. The argument for this claim was borrowed from Odum [4]: stability increases with the number of paths through which energy can flow up in a food web. Later, Elton [5] provided a suite of arguments for this positive relationship. The first one is the following: mathematical systems composed of one predator and one prey exhibit conspicuous fluctuations. Implicit in this argument is that more complex systems should be more stable, which remained untested at that time. In the seventies, Levins [6, 7], Ashby and Gardner [8], and May [9] showed numerically that large random systems may be expected to be stable up to a certain connectance threshold, contradicting the earlier ideas that complex natural systems are more likely to be stable. In his impactful work, May [10, 11] showed mathematically using random matrix theory [12, 13] that large and random ecosystems are inherently unstable. His approach was based on a mathematical study of community matrices, which represented unstructured random networks of interacting species. He used a local-stability analysis assuming these systems were at equilibrium. He derived a simple and elegant criterion for system stability, which is a milestone in the stability-complexity debate [14]. May [11] concluded that there was no comfortable theorem assuring that increased complexity will lead to stable systems, and that the task was therefore to “elucidate the devious strategies which make for the stability in enduring natural systems”.

Recently, the work of May was revisited by Allesina and collaborators [15, 16]. They established stability criteria for systems where species interact specifically via either competition, mutualism, or predation. As such, their contribution is a refinement of May’s approach that considered mixtures of interaction types. The general conclusion is that, in species-rich communities, interactions should be moderate to ensure the stability of the equilibrium. They also performed simulations to study stability in randomly generated systems whose architecture mimics empirically observed food webs [17, 18]. They arrived at the counter-intuitive conclusion that such ecologically-inspired structured graphs are less stable than unstructured ones. They also focused on interaction weights, and interestingly found that weak interactions should increase the stability for mutualistic and competitive webs, but decrease the stability of food webs (see also [19], where more precise statements are obtained for interactions weights of different intensity and symmetry). It was also found that more realistic structures seem to be detrimental for stability, and that the structure alone plays a minor role for stability compared to interaction weights [20, 21].

**Fig 1:**
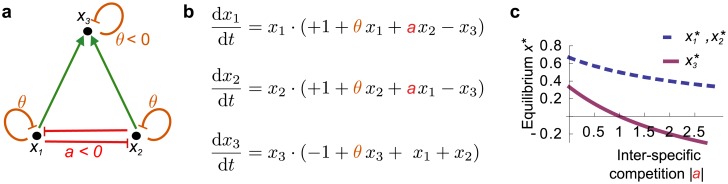
Feasibility and stability for a two-level food web. (**a**) The network is composed of two producers and of one consumer. (**b**) The time evolution of the species abundances is described by a Lotka-Volterra dynamic. The parameters defining the dynamical system are the interspecific competition coefficient *a* < 0 and the intraspecific competition coefficient *θ* < 0. The producers’ growth rates are set to 1, while the consumer growth rate is set to −1. (**c**) Abundances at equilibrium as a function of |*a*| with *θ* = −1. The system possesses a unique stable equilibrium which is feasible when |*a*| is smaller than a critical threshold. When |*a*| crosses this threshold, the stable equilibrium becomes infeasible.

**Fig 2:**
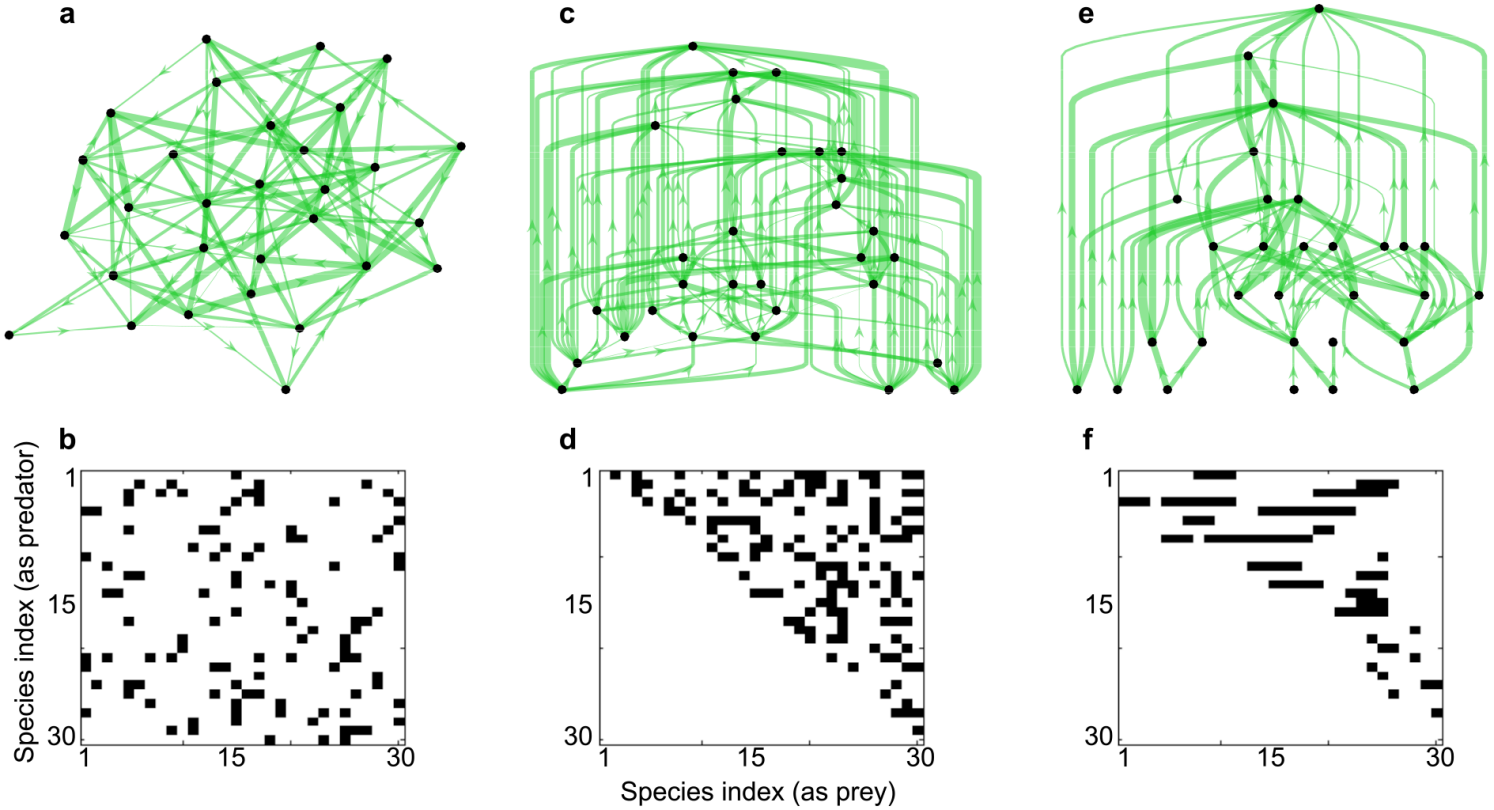
Illustration of some predator-prey networks and adjacency matrices. (**a**-**b**) Unstructured model for predation. (**c**-**d**) The cascade model. (**e**-**f**) The niche model. The parameters are *C* = 0.25 and *S* = 30. Thicker green arrows represent larger interaction strengths. Only positive interactions are represented.

All the above approaches are local-stability analyses, where systems are linearised at the equilibrium point and stability is evaluated only in the close vicinity of this point. In ecological systems, this point is meaningful only if the equilibrium-abundances are all strictly positive; in other words, if the equilibrium is feasible. Fig 1 illustrates the fact that a stable equilibrium of a simple two-level food web can be feasible when the absolute value of the interspecific competition coefficient is small, but infeasible when it becomes too large. Hence, a stable and feasible equilibrium can be transformed into an infeasible stable equilibrium by changing competition coefficients. In these previous works, equilibria were simply assumed feasible, without further analysis; basically, the true Jacobian, evaluated at the equilibrium, was replaced by a random matrix. Interestingly, soon after the work of May [11], Roberts [22] noted that May’s approach indeed remained silent concerning the feasibility of the equilibria. In a simulation study, he found that, in feasible systems, stability increased with the number of species. However, Roberts did not explore the very question of the relationship between system size and feasibility probability. This is a key issue since feasibility is a prerequisite to local-stability analysis. In the particular case of competitive systems, this question had to await the work of Logofet [23] who found that “equilibriumness” was vanishing with species richness. Note that already in 1970, Vandermeer [24] studied a question very related to feasibility, the expected number of species that can coexist in competitive communities. The same question has been treated by Rossberg [25], where the coefficient of variation of species abundance is studied in a mean-field approach. Since then, few contributions have explored the question of feasibility, and if so in particular systems and with regard to the characteristics of the interactions rather than to species number (e.g., for asymmetric competition [26, 27] or for mutualism [28]). Very recently the question of feasibility reappeared with the aim of providing general criteria leading to feasible equilibria [29], following an analogue approach as in ref. [28]. The idea of this important work is to compute the fraction of demographic parameters leading to feasibility under given conditions. Very interestingly, from the perspective of system complexity whose effect on stability has been thoroughly investigated in previous works [4, 6, 8, 9, 15], keeping this fraction well-defined (different from zero and infinity) when the number of species increases requires that the absolute mean interaction strength is small enough [29, Fig 2].

Here, we present an extensive study of the fundamental question of equilibrium feasibility, and expose its underlying mathematical principles in order to investigate how complexity influences feasibility probability. Two classes of networks are studied: 1) networks which do not possess any particular topological structure, for which we consider random, mutualistic, competitive, and predator-prey interactions, and 2) predator-prey networks designed to capture food web architecture, following the cascade [17], niche [18] and nested-hierarchy [30] models. We show that, in situations compatible with the above works [10, 15, 16], the existence of a feasible equilibrium is not guaranteed, since the probability to observe such a point decreases exponentially towards zero as the size of the network grows. Interestingly, we find that with weak interactions, non-trivial feasible equilibria are found only in ecologically-inspired systems, in a way that is ecologically sensible.

## Models

We adopt the following strategy: for models of interacting species, we use the demographic parameters most favourable to feasibility, and randomise the interactions to estimate the probability to find equilibria with only positive abundances. This is achieved for different species richness, network architectures, and interaction intensities. Additionally, in cases where the equilibrium is trivially feasible, we extend our analyses by investigating the effect of the demographic parameters on feasibility. We provide analytical results for cases compatible with May’s approach, and otherwise rely on simulations.

We consider a classical Lotka-Volterra model [31] for large, complex ecological networks with *S* species. Mathematically, in this setting, the vector of abundances *x* solves the following system of differential equations

$$\frac{dx_{i}}{dt}=x_{i}(r_{i}+\theta x_{i}+\frac{1}{{\left(CS\right)}^{\delta }}\sum_{j=1}^{S}a_{ij}\;x_{j}),\;\;\text{for}\;\text{all}\;1\leq i\leq S.$$

The per capita effect of species *j* on species *i* is encapsulated in a coefficient *a*_*ij*_, *r*_*i*_ denotes the intrinsic growth rates of species *i*, *θ* is a coefficient reflecting intraspecific competition, *C* is the connectance and *δ* a normalisation parameter (see below). In matrix notation, it becomes

$$\dot{x}=x\circ \left(r+\left(\theta I+{\left(CS\right)}^{-\delta }A\right)x\right)$$

with *I* the *S* × *S* identity matrix, and where ∘ denotes the product defined by *x* ∘ *y* = (*x*_1_*y*_1_,…,*x*_*S*_*y*_*S*_).

### Web topologies

We will first focus on webs whose topologies are based on Erdös-Renyi random graphs (unstructured webs), where edges occur with probability *C*, the so-called connectance of the graph. It is given by *C* = *L*/(*S* ⋅ (*S* − 1)) with *L* the total number of links in the network. Next, we will consider graphs whose topologies are drawn randomly according to the cascade [17], the niche [18] and the nested-hierarchy food web models [30] (structured webs), that capture more accurately trophic interactions of real food webs. Fig 2 provides illustrations of these various topologies.

### Interaction sign

If species *i* and *j* interact; that is if the web contains an edge between these two species, the nature of the interaction is encoded in the sign of the coefficients *a*_*ij*_: *a*_*ij*_ < 0 and *a*_*ji*_ < 0 for competition; *a*_*ij*_ > 0 and *a*_*ji*_ > 0 for mutualism; *a*_*ij*_ > 0 and *a*_*ji*_ < 0 for predation of *i* on *j*. The interactions *a*_*ij*_ form the *S* × *S* interaction matrix *A*.

In order to exhibit the effects of network structure on the dynamics, the *a*_*ij*_s are randomised with fixed mean and standard deviation. Their magnitude plays a key role for the local stability of networks [32, 33].

### Interaction strength

**Fig 3:**
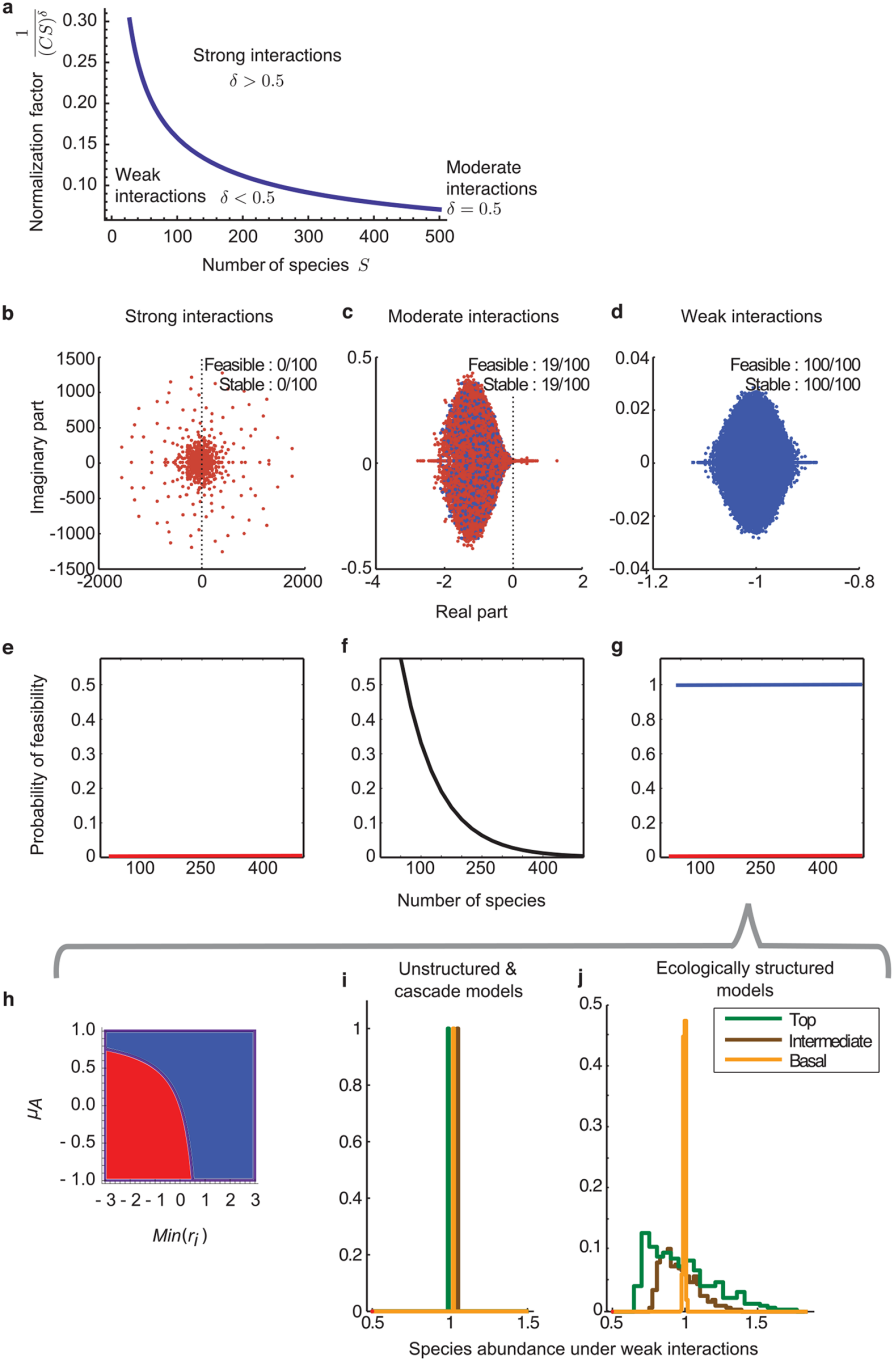
Stability and feasibility: Outline. (**a**) For a system to be feasible, interaction strength has to decrease with complexity. The Y-axis represents an interaction-strength normalizing factor that defines three regimes depending on the parameter *δ*: strong interactions (*δ* > 0.5), moderate interactions (*δ* = 0.5; blue line, with *C* = 0.4), and weak interactions (0 ≤ *δ* < 0.5). (**b, c, d**) For the three regimes, the graphs show the eigenvalues of the Jacobian *J*(x*) for 100 realisations of May’s random model (*S* = 150, *θ* = −1, *σ* = 0.4). Eigenvalues with a real part larger than 0 are unstable; eigenvalues of feasible systems are in blue. (**b**) With strong interactions, equilibria are never feasible nor stable. (**c**) With moderate interactions, feasible equilibria are stable. (**d**) With weak interactions, all equilibria are feasible and May’s stability criterion is trivially fulfilled. (**e, f, g**) Probability of feasibility for the three regimes: null under strong interactions; decreasing under moderate interactions; null or one under weak interactions. (**h**) In the weak regime, the probability of feasibility depends on the parameter values, for example the mean interaction strength (*μA*) and minimal growth rates (*Min*(*r*_*i*_)). The panel shows the result of Theorem S.3.6 for *θ* = −1 and $\bar{r}=1$. Blue region: parameters values leading to feasible equilibria (in red, to unfeasible equilibria). Note the trade-off between *μA* and *Min*(*r*_*i*_). (**i, j**) Under weak interactions, unstructured networks and the cascade model lead to deterministic abundances at equilibrium (in our setting, prey, predator, and intermediate species reach equal abundances). Interestingly, in structured networks (as the niche and the nested-hierarchy model), the abundances at equilibrium remain random, with a support depending on the trophic position of the species in the web.

We introduce a parameter 0 ≤ *δ* ≤ 1 ruling the average interaction strength. The *a*_*ij*_s are divided by the linkage density (a measure of complexity given by *CS* [10]) raised to the power *δ* (see ref. [28, 34]). This normalisation is mathematically sensible since Wigner’s theory [12], on which May’s and Allesina’s results are based, is built for the case *δ* = 1/2. Analytically, we will show that three regimes emerge when *S* becomes large: strong (0 ≤ *δ* < 1/2), moderate (*δ* = 1/2), and weak interactions (1/2 < *δ* ≤ 1), see Fig 3a. Intraspecific competition is included as customarily [10, 11, 15] with a common coefficient *θ* < 0, separated from the interaction matrix and unaffected by the normalisation constant.

### Construction of the networks

The first type of networks (unstructured) are based on Erdös-Renyi graphs. For May’s random model [10], an entry of the adjacency matrix *α* is set to one with probability *C*, zero otherwise. For mutualistic, competitive and predator-prey interactions, a pair (*i*, *j*), *i* < *j*, is linked such that *α*_*ji*_ = *α*_*ij*_ = 1 with probability *C*. The adjacency matrices for the second type of networks (structured networks modeling predation) are built according to the cascade [17], niche [18] and nested-hierarchy [30] models. Their adjacency matrix *α* indicates that *i* preys upon *j* when *α*_*ij*_ = 1 and their connectance is set to *C*/2. Their detailed construction is reported in S1 Text. See also Fig 2 for an illustration.

In May’s random model [10], interactions strengths are sampled from i.i.d. random variables, leading to mixture of interactions types, including also commensalism and amensalism. Concerning models for mutualism (competition), *a*_*ij*_ and *a*_*ji*_ are independently randomly chosen from a positive (negative) random variable. For predation models, any pair (*i*, *j*) such that *i* < *j* is linked (i.e. *α*_*ji*_ = *α*_*ij*_ = 1 in the adjacency matrix) with probability *C* and the interactions strengths are also independently sampled, with the restriction that sign(*a*_*ij*_) = −sign(*a*_*ji*_). For the structured networks, the entries of *α* are multiplied with positive i.i.d. values and the entries of *α*^*t*^, the transpose of *α*, with negative i.i.d. values. The interaction matrix *A* is the addition of the two. Gaussian or folded-Gaussian random variables with standard-deviation *σ* are chosen in our simulations, but our analytical results are not limited to the particular choice of these distributions, see S1 Text. Our simulations are performed by randomly sampling *A* and then computing the equilibrium x* according to Eq (1). This Monte-Carlo procedure is used for the estimation of *P*_*S*_ under the different scenarios.

## Results

### Existence of feasible equilibria

A system is feasible if all abundances at equilibrium are positive. If this equilibrium exists, the vector x* corresponds to a point for which the dynamics of the Lotka-Volterra system stops varying, and is given by

$$x^{*}=-{\left(\theta I+{\left(CS\right)}^{-\delta }A\right)}^{-1}r\;.$$ (1)

If the solution to this equation involves negative abundances, then the system is not feasible. Since the interactions *a*_*ij*_ are random variables, it is natural to study *P*_*S*_, the probability to observe a feasible equilibrium, which is given by

$$P_{S}=P\left(x_{i}^{*}>0,\;\text{for}\;\text{all}\;\text{species}\;i\right).$$ (2)

This probability depends on *θ*, *C*, *S*, *δ*, *A* and *r*.

For a given *δ*, *C*, and type of network, we choose the growth rates vector *r* = (*r*_*i*_) most favourable for feasibility, or, in case of trivially feasible equilibria, *r*_*i*_ to be independent and bounded random variables. The most favourable growth rates correspond to the deterministic vector making the equilibrium to be, on average, as far as possible from the boundaries of the feasibility domain. This deterministic vector is referred to as the mean structural vector. By definition, the feasibility domain is the positive orthant of the phase space. We show in the S1 Text that this vector is related to a particular way of choosing intrinsic growth rates, which have been used in ref [20] to avoid negative abundances.

It is now possible to compute *P*_*S*_ with regard to the randomness of the *a*_*ij*_s. Extending results on large systems of random equations [35], we develop analytical formulas for *P*_*S*_ for our first class of unstructured models when *S* tends towards infinity, and use Monte Carlo simulations to estimate *P*_*S*_ for the second class of structured models (see S1 Text). Before exposing our results, we briefly discuss the link between stability and feasibility.

### Link between stability and feasibility in random models

It is only when *A* is such that x* is feasible that the local-stability analysis of x* is sensible. This analysis involves the Jacobian matrix evaluated at x* (the so-called community matrix),

$$J\left(x^{*}\right)=\text{diag}\left(x^{*}\right)(\theta I+{\left(CS\right)}^{-\delta }A),$$ (3)

which of course depends on the random vector x* and on the random interactions matrix *A*. The equilibrium x* is linearly stable when the real parts of all eigenvalues of *J*(x*) are negative. Under May’s approach, and similarly in ref. [15, 16, 19], *J*(x*) is replaced by the random matrix $\tilde{J}=\theta I+{\left(CS\right)}^{-\delta }A$. All the information on x* and on the inherent relations between x* and *A* (see Eq (1)) are overlooked. The problematic point is that one obtains stability results by focusing on the eigenvalues of $\tilde{J}$ even when a feasible equilibrium x* does not exist.

For example, May [10] considers that the elements of *A* are such that all diagonal entries are set to 0, and that all off-diagonal random elements are independent and set to 0 with probability 1 − *C*. A non-zero entry is sampled from any centred distribution with standard deviation *σ*. Applying results from random matrix theory [13], the eigenvalues of $\tilde{J}$ will then have negative real parts for large *S* when

$$\frac{\sigma }{{\left(CS\right)}^{\delta }}\sqrt{CS}<\left|\theta \right|,$$ (4)

which is May’s stability condition. We show in S1 Text, Proposition S.4.1, that May’s criterion still holds for the matrix *J*(x*) under the additional assumption that x* is feasible (see Fig 3b). Thus it appears critical to study the feasibility of such systems.

### Feasibility in unstructured random web models

#### Strong interactions

For 0 ≤ *δ* < 1/2, the probability of finding feasible equilibria *P*_*S*_ goes abruptly to zero. Indeed, the variance of the abundances at equilibrium of each species grows with species richness. Then, the probability of having negative abundances converges to one, consequently *P*_*S*_ → 0, as illustrated in Fig 3c. Note also that this regime of interactions violates May’s stability criterion when *S* increases.

#### Moderate interactions

Moderate interactions (*δ* = 1/2) correspond to the limiting case for which May’s criterion can be asymptotically satisfied. Indeed, from Eq (4), May’s stability criterion is now independent of *S* and *C*, and becomes *σ* < |*θ*|. However, we show that in this case there exists asymptotically almost surely no feasible equilibria. Indeed, we prove that, for independent and identically distributed (i.i.d.) intrinsic growth rates *r*_*i*_ and when *σ* < |*θ*|, the equilibrium abundances $x_{i}^{*}$ are asymptotically i.i.d. Gaussian random variables

$$x_{i}^{*}\approx N(\mu_{*},\sigma_{*}^{2})\;\text{for}\;\text{all}\;\;i=1,\ldots ,S,$$

where the mean and the variance are given by

$$\mu_{*}=-\frac{\bar{r}}{\theta }\;\;\text{and}\;\;\sigma_{*}^{2}=\frac{\sigma_{r}^{2}}{\theta^{2}}+\frac{r^{2}\sigma^{2}}{\theta^{2}\left(\theta^{2}-\sigma^{2}\right)},$$

(see Proposition S.3.2. of S1 Text). In the above formula, $\bar{r}$, $\sigma_{r}^{2}$ and *r*^2^ denote respectively the mean, the variance and the second moment of the random intrinsic growth rate *r*_*i*_. When *σ* ≈ |*θ*|, the system becomes structurally unstable (see [36]): the random matrix *θI* + (*CS*)^−*δ*^ *A* becomes singular and the linear system (1) is ill-conditioned, that is, the inverse of the random matrix is dominated by large contributions associated with near zero eigenvalues.

**Fig 4:**
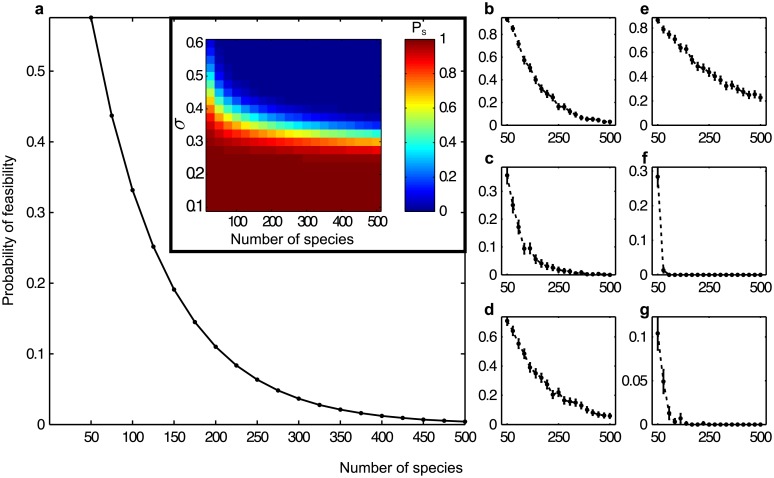
Probability of feasibility for different models in the regime of moderate interaction strength. (**a**) Analytical prediction for the random model. Insert: the scaled colors express *P*_*S*_ as a function of both *S* and *σ*. (**b-g**) Predictions (with 95% confidence intervals) from 1000 simulations for random mutualistic networks, random competitive networks, random predator-prey networks, the cascade model [17], the niche model [18], and the nested-hierarchy model [30], respectively. We choose $a_{ij}∼N\left(0,\sigma \right)$ for any non-zero entry of the interaction matrix *A* in the random model; in the other cases, a strictly positive interaction is randomly drawn from a folded normal distribution such that $a_{ij}∼|N(0,\sigma )|$, and a strictly negative interaction is sampled such that $a_{ij}∼-|N(0,\sigma )|$. The parameters are *C* = 0.25, *σ* = 0.4, and *θ* = −1.

By independence of the $x_{i}^{*}$, the probability of feasibility $P_{S}=P\left(x_{i}^{*}>0,\;\text{for}\;\text{all}\;\text{species}\;i\right)$ reduces thus to $P_{S}=\prod_{i=1}^{S}P\left(x_{i}^{*}>0\right)$ in the large *S* limit. Since every $x_{i}^{*}$ is identically normally distributed, $P(x_{i}^{*}>0)<1$ for every 1 ≤ *i* ≤ *S*. Thus *P*_*S*_ decreases following a power law when the number of species *S* becomes large, i.e. *P*_*S*_ → 0 as *S* → ∞ (Figs 3c and 4a. See Theorem S.3.3. in S1 Text for complete details). More precisely, when the intrinsic growth rate is the mean-structural vector, the probability that an equilibrium is feasible is approximated by

$$P_{S}\approx \Phi (\sqrt{\frac{\theta^{2}-\sigma^{2}}{\sigma^{2}}}{)}^{S},$$ (5)

where Φ is the standard Gaussian cumulative distribution function. Two parameters influence the decrease of *P*_*S*_; the intraspecific competition *θ* and the variance of the interactions *σ*^2^. Larger *θ* will increase Φ (which becomes closer to one) and reduce the speed of decrease of *P*_*S*_. Concerning the interactions, smaller variances permit a similar behavior, as illustrated in the insert of Fig 4a. In this illustration, standard-deviations smaller than *σ* = 0.3 produce more likely feasible equilibria in systems with finitely many species.

Concerning local stability within this framework, interestingly, we prove in Proposition S.4.1 that any asymptotically feasible equilibrium is stable when *σ* < |*θ*|.

We also show numerically that similarly *P*_*S*_ → 0 as *S* → ∞ for unstructured random models for competitive, mutualistic, and prey-predator networks (see Table 1, Fig 4, and S1 Text).

**Table 1:** Summary of the different results presented on *P*_*S*_ for *S* → ∞.

Model		Moderate interactions	Weak interactions
		($\delta =\frac{1}{2}$)	($\frac{1}{2}<\delta \leq 1$)
Unstr.	May’s model	*P*_*S*_ → 0	*P*_*S*_ → 0 or 1
	Competition	*P*_*S*_ → 0	*P*_*S*_ → 0 or 1
	Mutualism	*P*_*S*_ → 0	*P*_*S*_ → 0 or 1
	Predation	*P*_*S*_ → 0	*P*_*S*_ → 0 or 1
Struct.	Cascade	*P*_*S*_ → 0	*P*_*S*_ → 0 or 1
	Niche	*P*_*S*_ → 0	x* not deterministic and *P*_*S*_ → 0 or 1
	Nested hierarchy	*P*_*S*_ → 0	x* not deterministic and *P*_*S*_ → 0 or 1

The probability *P*_*S*_ converges towards 0 as *S* → ∞ for moderate interactions (and with the mean structural vector) in unstructured (unstr.) and structured (struct.) networks. For weak interactions, *P*_*S*_ → 0 or *P*_*S*_ → 1, depending on the parameters. The equilibrium x* is deterministic in the unstructured case and for the cascade model. x* has a non-trivial distribution and is feasible with positive probability for the niche and nested-hierarchy models.

#### Weak interactions

**Fig 5:**
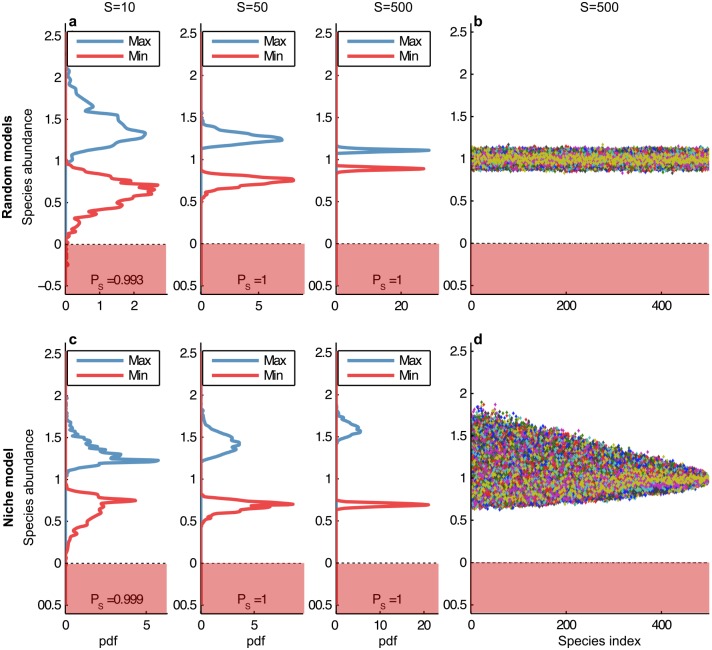
Convergence of the equilibrium under weak interactions. For *S* = 10, 50, and 500 the distribution of max(x*), in blue, and min(x*), in red, are represented for 1000 simulations for (**a**) random networks, and (**c**) the niche model. For *S* = 500, all simulations are plotted to illustrate the support of the distribution of x*: (**b**) in random networks, the regime of weak interactions yields asymptotically deterministic equilibria; (**d**) in the niche model, the equilibria are no longer deterministic and converge towards a distribution with a non-trivial support. Species range from top predator (index *i* = 1) to basal (*i* = *S*); in (**d**), note that the size of the support depends on the index of the species.

Consider now weak interactions among species (1/2 < *δ* ≤ 1) in unstructured random networks. When the growth rate vector is set to the mean-structural vector, then *P*_*S*_ → 1 (Fig 5b). Indeed, we prove in S1 Text that the steady state x* converges towards the feasible deterministic vector 1 which contains all 1s as values. In other words, any $x_{i}^{*}$ converges to 1 with a variance converging to 0 and is thus trivially positive. This implies *P*_*S*_ → 1.

We test now different growth rates. Considering a general model where *μ*_*A*_ denotes the mean interaction strength between interacting species, we find that *P*_*S*_ → 1 when $r_{i}>\bar{r}\;\mu_{A}/\left(\mu_{A}+\theta \right)$ for all *i* and where $\bar{r}$ is the sample mean of the intrinsic growth rates (see Theorem S.3.6. of the S1 Text), otherwise, *P*_*S*_ → 0 as illustrated in Fig 3d. For example, in May’s framework (*μ*_*A*_ = 0), *P*_*S*_ → 1 when all growth rates are positive. Similar results will be found in S1 Text concerning other types of unstructured models. These results rely on the fact that, when the growth rate vector has bounded components, the variance of any $x_{i}^{*}$ goes towards zero. As such, x* converges towards a deterministic vector that is feasible or not depending on the chosen parameters values. Thus *P*_*S*_ → 1 or 0, depending on *r*_*i*_, *θ*, and *μ*_*A*_.

Overall, with weak interactions and for any choice of growth rates, all realisations of the random matrix *A* lead to a limiting deterministic equilibrium. Thus, abundance variability disappears irrespective of particular realisations of *A*.

When studying the three other types of unstructured models (mutualistic, competitive, and predator-prey networks), we find the same results as above for strong, moderate or weak interactions (Table 1).

### Feasibility in predator-prey structured models

May’s paradox that complexity decreases stability in mathematical models led to the exploration of the effects of topological structure on systems’ dynamics. Several works have shown that particular architectures can stabilise ecological networks [37–40]. Nevertheless, the feasibility of different network architectures for different interaction regimes has not been explored.

Consider for example Cohen’s cascade model of trophic networks [17], where species are hierarchically ordered so that they feed only on lower indexed species, generating sub-triangular matrices with random structure. For the three regimes of *δ*, simulations yield similar behaviour of *P*_*S*_ as for unstructured models (Table 1).

**Fig 6:**
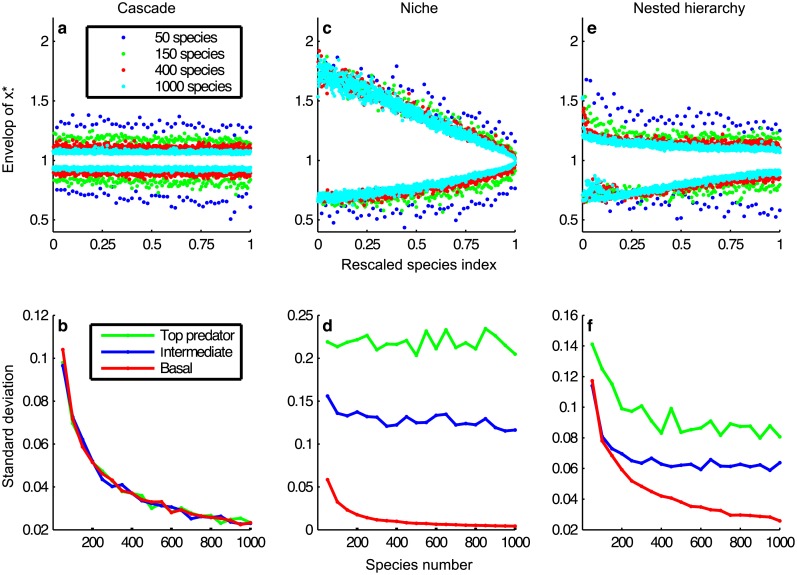
Behaviour of the structured models under weak interactions with the mean structural vector. In the first row, we plot for different values of *S* the envelop (the maximum and the minimal value) of the equilibrium x* among 1000 simulations. The species have been assigned a number between zero (top species) and one (basal species) corresponding to their hierarchy in the web. In the second row, the standard deviations of $x_{i}^{*}$ for *i* = 1 (top predator), *i* = *S*/2 (intermediate) and *i* = *S* (basal) are represented as a function of *S*. The parameters are *C* = 0.25, *σ* = 0.4 and *θ* = −1. (**a** and **b**) In the cascade model, although hierarchically ordered, species tend to behave similarly with regard to convergence as *S* grows. The equilibrium converges almost surely to the vector 1. (**c** - **f**) The niche and the nested-hierarchy models keep the randomness of the equilibrium when *S* grows, even under weak interactions.

We now test two other food web models that more accurately capture the structure of trophic interactions in real systems, the niche [18] and the nested-hierarchy models [30]. The former generates purely interval food webs (predators consume all species in a niche interval) while the latter is based on evolutionary processes and relaxes this constraint. The results are qualitatively similar to the ones of unstructured random models for all types of interactions. However, two interesting features emerge. First, the mean structural vector now contains negative growth rates, which is the essence of predator-prey systems where predators will die in the absence of prey (this feature also appears in the cascade model, but not in the unstructured food web model, see Table 2 in S1 Text). Second, with weak interactions, each $x_{i}^{*}$ no longer converges to a deterministic limiting vector, but instead to a non-trivial random variable having a distribution of finite support and positive standard-deviation. Thus, contrary to the unstructured and cascade models, a particular realisation of the interaction matrix *A* will influence the equilibrium values. Indeed, these highly structured networks and their related mean structural growth rates induce correlations among the abundances at equilibrium, which prevent a convergence to a deterministic value. We find that the size of the support depends on the hierarchical position of the species (Fig 6). We performed additional simulations based on empirical food webs, and find similar results (Figs G and H in S1 Text).

**Fig 7:**
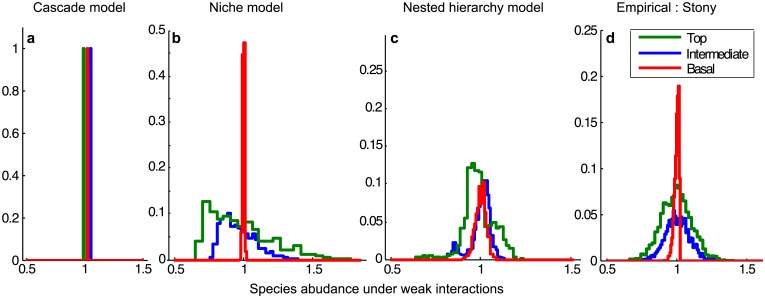
Empirical distribution of top, intermediate and basal species under a regime of weak interactions. (**a**) Cascade model with *S* = 800. (**b**) Niche model with *S* = 800. (**c**) Nested-hierarchy model with *S* = 800. The same parameters as in Fig 6 have been used among 1000 simulations. (**d**) The distribution of a top, intermediate and basal species from the empirical food web Stony is illustrated (see S1 Text). The interactions strengths have been simulated similarly as for any structured web. The mean structural vector has been simulated with Monte-Carlo methods (200 trials).

Since the mean structural vector is constructed for the purpose of maximizing the probability of feasibility, we have also considered random Gaussian growth rates of positive mean to complete our analysis. For the niche and nested-hierarchy models, the standard deviations $\sigma_{i}^{*}$ of the abundances at equilibrium are ordered according to the trophic position of the species within the food web (from basal autotrophs, to intermediate species that are predator and prey, and to top predators). We find $\sigma_{\text{basal}}^{*}<\sigma_{\text{intermediate}}^{*}<\sigma_{\text{top}}^{*}$, a result similar to that obtained with the structural vector, see Figs 3e and 7. This effect, also visible in empirical food webs (Figs G and H in S1 Text), disappears in two particular cases: 1) for zero mean Gaussian random growth rates; 2) when the growth rate standard-deviation becomes large. In both cases, the equilibria then become independent of species trophic position (Figs G and I in S1 Text).

## Discussion

Our results can be summarised as follows: for the cases compatible with May’s [10] and Allesina’s [15, 16] framework where *δ* = 1/2 (moderate interactions), there exist almost surely no feasible equilibria in species-rich systems for any of the models considered. For weak interactions in randomly structured systems, feasibility is granted; also, the stability criteria are trivially asymptotically satisfied. Therefore, in such situations, the criteria are basically void of information. However, including realistic structure in trophic systems, we find that the results are no longer trivial.

As a general message, future work tackling dynamical stability must be preceded by a feasibility analysis. In our Lotka-Volterra framework, this involves knowledge of the structure and intensity of interactions, and of the growth rates. Feasibility can then be easily evaluated. Our results indicate that sensible information on the dynamics of natural systems will be obtained by concentrating on the interplay between ecological structure, strengths of interaction weights, and how interaction strengths are distributed in their architecture.

There are still many other aspects that should be taken into account to reach general conclusions about feasibility and stability that better fit natural systems. They are very different from unstructured systems, and many constraints affect their architecture and dynamics. A first question simply concerns system size in terms of species richness *S*. Most described systems are middle sized, but large webs do exist [41, 42], so that mathematical study focusing on large *S* limits can be justified. An intriguing aspect here is how smaller and well-definable webs are dynamically embedded in larger species-rich systems. For example, it has been found that top predators have a stabilising effect on food webs by coupling fast and slow energy channels in natural aquatic systems [43]. This question could be framed in a general theory for the “inverse pyramid of habitat”, which explores the consequences of the ubiquitous (but often overlooked) observation that species located at higher trophic levels tend to have larger home ranges [44]. In this respect, the spatial structure of the system is relevant and has non-trivial consequences for its dynamics [45, 46], a research framework that is currently expanding (e.g., [47–49]).

Relevant to system size lies also the question of how network nodes are defined: taxonomic resolution is often heterogeneous in observed food webs, with basal species being more often pooled (e.g., phytoplankton being considered as a single species). Secondly, species richness *S* and connectance *C* are usually treated as independent parameters, while it has been described that *C* decreases with *S* in different systems [50], thus restricting the parameter space to be explored. Finally, even if topology per se has been suggested to play a minor role in system stability [20], we showed that network architecture does play a non-trivial role on equilibrium abundances. Layer architectures as those produced by the niche and nested-hierarchy models yield ecologically sensible results; exploring other structural models [21, 51–53] and especially natural architectures is necessary.

Apart from these structural considerations, ecologists are confronted by a more elusive issue, the estimation of demographic parameters. To make sense, the investigation of feasibility and stability should be based on meaningful parameter values. The growth rate vector *r* that appears in Lotka-Volterra dynamics is mostly unknown or very difficult to obtain experimentally. One can either keep this vector as an intrinsic free parameter or assume that such growth rates change with interaction weights, as in [20] or with our mean structural vector. The estimation of interaction parameters is also far from trivial [54–57]. Experimental studies (see, e.g., [20, 58, 59]) choose interaction weights indirectly, according to particular methods like predator-prey mass ratio models, biomass flux, or the Ecopath method (see, e.g., [60–62]). Additionally, one unresolved question is how intraspecific competition scales with average interaction strength, which is perhaps the most important ingredient for stability (e.g., [10, 15, 58, 63]), and also plays a role in feasibility (see Proposition S.3.2 in S1 Text). Also, if the magnitude of intraspecific competition can be assumed to be independent of *S*, this is likely not the case with interspecific interactions (captured by our parameter *δ*), which has been shown to play a key role in mutualistic networks [28]. This question is related to the study of weak interactions, which have been shown to promote stability [19, 32, 58, 59] and here feasibility. Finally, one must consider that these parameters do scale with body size [64], which defines a constraint between parameter values and network structure, as large-bodied species typically populate higher trophic levels. Interestingly, this allometric relationship may underlie the result that stability depends on the link between trophic position and interaction strength [20], and our finding that abundance variability scales with trophic position.

For mathematical tractability, many studies on the dynamics of food webs, including ours, rely on Lotka-Volterra models with mass-action type interactions (the so-called Holling type I functional response). However, investigations on the number of prey eaten per predator and unit time in simple systems do not support this modelling assumption. There exists a vast literature on the subject and the choice of a sensible functional response is still debated [65, 66]. In Section S.5 of the S1 Text, we studied a predation unstructured model with a Holling type II functional response. We found, independently of the choice of the regime *δ*, that the system behaves similarly to the Lotka-Volterra model with weak interactions. Here, the saturation inherent to Holling type II functional response makes interactions weak, irrespectively of the parameter *δ*. It shows that interaction dynamics can strongly affect system feasibility in predator models. Other aspects related to interspecific interactions affect community dynamics, e.g., evolving trait-mediated direct and indirect interaction weights [67–69], ontogenetic shifts in interactions [70], or high-order interactions whereby the interaction between two species is modulated by one or more other species [71]. Also, it has been shown that “foraging adaptation” by predators affects the dynamics of food webs [72]. Such non-linearities in species interactions have been explored from the stability point of view of systems adjusted to be feasible [73–75]. However, since the precise way interactions are modelled has non-trivial consequences on system dynamics, the relationship between feasibility and complexity should also be considered in such situations.

May’s approach considered systems with any kind of interaction and not only trophic ones. An articulate answer on the relationship between stability and complexity should obviously incorporate all types of interactions relevant for system dynamics (including the effects of ecosystem engineers [76, 77]). This question has been the focus of recent theoretical developments [78, 79], and empirical studies specifically addressing this question start to emerge [80].

Science faces increasingly complex situations where high-dimensional parameters occur. A good example is statistical mechanics, for which relevant results have been obtained for highly complex systems without having a precise knowledge of the microscopic details of a model. Our results like many cited here follow the same line. However, due to the complexity of natural systems, the gap between theoretical and empirical investigations is likely to remain open. To attain a consensus, the feasibility of the model systems should not be forgotten [23, 24, 26–29], and more effort must be devoted to obtaining empirical and experimental time series as necessary benchmarks.

## Supporting information

S1 Text Supplementary text. Specific methods, details, definitions and proofs of the results.
(PDF)
