# Supplementary material for: The feasibility of equilibria in large ecosystems: A primary but neglected concept in the complexity-stability debate
Source: PLoS Comput Biol. 2018 Feb 8;14(2):e1005988. doi: 10.1371/journal.pcbi.1005988 (PMC5821382; doi:10.1371/journal.pcbi.1005988)
Supplement: S1 Text — Specific methods, details, definitions and proofs of the results. (PDF) [file pcbi.1005988.s001.pdf]

Supplementary Information of The feasibility of equilibria in large ecosystems: a primary but neglected concept in the complexity-stability debate

Michael Dougoud[1], Laura Vinckenbosch[1,3], Rudolf Rohr[2], Louis-Félix Bersier[2], and Christian Mazza[1]

[1] *University of Fribourg, Department of Mathematics, CH-1700 Fribourg*
[2] *University of Fribourg, Department of Biology, Unit of Ecology and Evolution, CH-1700 Fribourg*
[3] *University of Applied Sciences Western Switzerland-HES-SO HEIG-VD, CH-1401 Yverdon-les-Bains*

January 26, 2018

Contents

S.1 Summary of the results **2**

S.2 Model and methods **2**
S.2.1 Feasibility and stability in dynamical systems 2
S.2.2 Lotka-Volterra model 3
S.2.3 Interactions 4
S.2.4 Growth rates 7

S.3 Probability of feasibility **7**
S.3.1 Moderate interactions 9
S.3.2 Weak interactions 12
S.3.3 Empirical networks 17
S.3.4 Connectance and interactions strengths 17

S.4 Consequences on local stability analysis **19**
S.4.1 Random networks 20
S.4.2 Structured models 23

S.5 Feasibilty with a Holling type II functional response **27**

Supplementary references **28**

S.1 Summary of the results

	Model	Moderate interactions ($\delta = \frac{1}{2}$)	Weak interactions ($\frac{1}{2} < \delta \leqslant 1$)
Unstructured	Random	$P_S \to 0$ (Thm. S.3.3)	$P_S \to 0$ or 1 (Thm. S.3.5)
	Competition	$P_S \to 0$	$P_S \to 0$ or 1 (Cor. S.3.8)
	Mutualism	$P_S \to 0$	$P_S \to 0$ or 1 (Cor. S.3.8)
	Predation	$P_S \to 0$	$P_S \to 0$ or 1 (Cor. S.3.9)
Structured	Cascade	$P_S \to 0$	$P_S \to 0$ or 1
	Niche	$P_S \to 0$	x^* not deterministic and $P_S \to 0$ or 1
	Nested hierarchy	$P_S \to 0$	x^* not deterministic and $P_S \to 0$ or 1

Supplementary Table A Summary of the different results presented on P_S for $S \to \infty$. The results are analytical if specified in brackets and otherwise obtained by simulations (see Sections S.3.1.2 and S.3.2.2 as well as Main Text).

S.2 Model and methods

S.2.1 Feasibility and stability in dynamical systems

Consider dynamics driven by a general system of first order autonomous ordinary differential equations

$$\frac{\mathrm{d}x_i(t)}{\mathrm{d}t} = f_i\left(x(t)\right), \quad i \in \mathcal{S} = \{1, \ldots, S\}, \quad t \geqslant 0,$$

with $f = (f_1, \ldots, f_S) : \mathbb{R}^S \to \mathbb{R}^S$ a differentiable function and $x(t) = (x_1(t), \ldots, x_S(t)) \in \mathbb{R}^S$. Solutions $x(t)$ of this system evolve in time and their trajectories are rarely describable as such, especially in high dimensions. However, there are some important features that can be exhibited without solving the system. One of the most instructive is the existence of *equilibria*, which are points x^* in the phase space $\mathbb{R}^S$ where the solution of the system does not vary in time, that is $f_i(x^*) = 0$ for all $i \in \mathcal{S}$. A trajectory reaching an equilibrium remains indefinitely at this point. If x^* belongs to the admissibility domain of the model (i.e., all $x_i^* > 0$ for ecological systems), then it is called *feasible*. Unfeasible equilibria are well-defined in the mathematical sense, but have to be rejected in the perspective of the application.

If small perturbations of the trajectory in the neighbourhood of the equilibrium x^* fade over time, so that the system tends to restore the equilibrium, then x^* is said to be *locally stable*.

This property is related to the derivatives of the function f. More specifically, an equilibrium x^* is *linearly stable* when the Jacobian matrix $J(x^*) = \left(\frac{\partial f_i}{\partial x_j}(x^*)\right)$ evaluated at the equilibrium has only eigenvalues with negative real parts (see [11], [67] or [68]).

S.2.2 Lotka-Volterra model

In the context of ecological networks, the dynamics of interacting species is commonly described by the Lotka-Volterra equations (see e.g. [69, 70, 29]). The function f can be written as

$$\frac{\mathrm{d}x_i}{\mathrm{d}t} = x_i \left(r_i + \theta x_i + \sum_{j=1}^{S} \frac{a_{ij}}{(CS)^\delta} x_j \right) = f_i(x), \; i \in \mathcal{S} = \{1, \cdots, S\}, \tag{S1}$$

where x_i denotes the abundance of species i, $\theta < 0$ is a friction coefficient (intraspecific competition, assumed to be the same for all species), r_i describes the intrinsic growth rate of i and the interaction coefficients a_{ij} stand for the per capita effect of species j on species i. The connectance C denotes the proportion of links present in the network with respect to the number of all possible links $S(S-1)$ or S^2, depending on the model. The product CS is a measure of the complexity of the system, and is the average number of links between two species. The term $(CS)^\delta$ is introduced as a normalisation of the interactions strength, with $\delta \geqslant 0$ a parameter controlling this renormalisation (see 2.3.3 below). In matrix form, the system writes

$$\frac{\mathrm{d}x}{\mathrm{d}t} = x \circ \left(r + \left(\theta I + \frac{A}{(CS)^\delta} \right) x \right), \tag{S2}$$

where I denotes the $S \times S$ identity matrix, $A = (a_{ij})_{1 \leqslant i,j \leqslant S}$ is the interaction matrix, $r = (r_1, \ldots, r_S)$ is the growth rates vector and $\circ$ denotes the Hadamard product, that is $x \circ y = (x_1 y_1, \ldots, x_S y_S)$.

The admissibility domain of this model is the positive orthant, that is $x \in \mathbb{R}^S$ with $x_i > 0$ for all i. A feasible equilibrium must then satisfy $x_i^* > 0$ for all species $i \in \mathcal{S}$ and

$$x^* = \left(-\theta I - \frac{A}{(CS)^\delta} \right)^{-1} r, \tag{S3}$$

so that the right hand side of (S2) equals zero, assuming this inverse matrix exists. Such an equilibrium is locally stable if the Jacobian matrix (also named community matrix in this context)

$$J(x^*) = \mathrm{diag}(x^*) \left(\theta I + \frac{A}{(CS)^\delta} \right) \tag{S4}$$

has all eigenvalues with negative real part. As one can see in the previous equation, this matrix depends explicitly on the equilibrium abundances x^* ($\mathrm{diag}(x^*)$ denotes the matrix with x^* in the diagonal and 0 everywhere else) and it should not be confused with the interaction matrix A.

Several ecological models have been developed with the goal of understanding network structure and its effects on system dynamics [71, 10, 17, 18, 28]. Those models generate essentially

different types of network structures, and consequently particular interaction matrices A. In order to exhibit the particular effects of a structure on the dynamics of systems, the coefficients a_{ij} can be randomised. This allows the exploration of some behaviour of the models and the detection of their key features. A direct consequence in considering random interactions is that feasibility and stability have to be considered from a probabilistic point of view. We define therefore the *probability of feasibility* of an equilibrium as

$$P_S = \mathbb{P}\left(x_i^* > 0\, \forall i \in \mathcal{S}\right). \tag{S5}$$

The purpose of this work is to study this probability, which expresses the likelihood of a model to provide feasible equilibria, for which the stability can eventually be examined.

S.2.3 Interactions

The interaction matrix A describes firstly who interacts with whom in the network (the structure), and secondly what is the type of these interactions: if $a_{ij} < 0$ and $a_{ji} < 0$, species i and j compete with each other; if $a_{ij} > 0$ and $a_{ji} > 0$, their interaction is mutualistic and they both benefit from the presence of each other; if $a_{ij} > 0$ and $a_{ji} < 0$, then species i preys upon species j; if $a_{ij} = 0$, species j has no direct effect on i.

The models of complex ecological networks that we consider can be divided into two main categories: unstructured and structured models. The former consist in webs for which the topological structure is completely free and random. This equates to considering networks based on Erdős-Rényi graphs, which are constructed by adding randomly edges between nodes with the same probability C. Here, we consider four of those models depending on the type of interactions: random (as in May's formalism [10]), mutualistic, competitive and predator-prey. Structured models define stochastic rules for the construction of the underlying graphs so that not all graphs are equally likely. This is the case in the three models studied here: the cascade [17], the niche [18] and the nested-hierarchy model [28]. Since these three models were built to represent food webs, only predator-prey interactions are considered. The section Method in the Main Text describes their construction in detail. Suppl. Fig. L provides an example of network samples for the unstructured predator-prey model and for the structured cascade and niche model.

S.2.3.1 Interaction models on unstructured networks

Random network. This model introduced by May in 1972 [10] considers both the structure and the type of interactions as fully random. The interactions are independent and identically distributed (i.i.d.) centred random variables with common standard deviation σ and can thus be either positive or negative, corresponding to a mixture of competition, mutualism and predation. The coefficients equal 0 independently of their position, with a fixed probability $1 - C$. Note that $a_{ij} = 0$ does not necessary imply $a_{ji} = 0$, so that amensalism $(0, -)$ and commensalism $(0, +)$ interactions are also possible. In this model, the connectance is given by $C = L/S^2$, where L is the number of links in the network.

Mutualistic network. This model considers only mutualistic interactions $(+, +)$. The diagonal coefficients are $a_{ii} = 0$ and any pair of species (i, j), $i < j$ is linked with probability C. If the

pair (i, j) is not linked, then $a_{ij} = a_{ji} = 0$, otherwise the interactions strength a_{ij} and a_{ji} are positive i.i.d. random variables. The elements of A are almost all i.i.d., except for the pairs a_{ij} and a_{ji}, which are only independent conditionally on the fact that they are different from 0. In this model, the connectance is given by $C = L/S(S-1)$.

Competitive network. This model is built in the exact same manner as the previous one, except that the interactions are nonpositive random variables in such a way that there are only competitive interactions $(-,-)$.

Predator-prey network. This is an example of an unstructured model for *predation*. The diagonal coefficients are $a_{ii} = 0$ and any pair of species (i, j), $i < j$ is linked with probability C. If the pair (i, j) is not linked, then $a_{ij} = a_{ji} = 0$, otherwise the interactions strength are independently sampled, with the restriction that $\text{sign}(a_{ij}) = -\text{sign}(a_{ji})$. This results in a sign antisymmetric interaction matrix whose elements are identically distributed and almost all independent, except for the pairs (a_{ij}, a_{ji}), which are correlated by their sign. The connectance in this model is given by $C = L/S(S-1)$.

S.2.3.2 Interaction models on structured networks

Cascade model. This model introduced by Cohen et al. [17] is an example of structured food webs. The interactions are of predator-prey type. The species are ordered on a line and they can feed only on species with a strictly lower rank, excluding any loop in the network. The resulting interaction matrix A has an upper diagonal with nonnegative i.i.d. entries and a lower diagonal with nonpositive i.i.d. entries. An upper diagonal entry $a_{ij}, i < j$ can be zero with probability $1 - C$ and in this case $a_{ji} = a_{ij} = 0$. The diagonal of A consists in zeros and the connectance is $C = L/S(S-1)$.

Niche model. In 2000, Williams and Martinez [18] proposed a new model that permits loops (including cannibalistic loops), and that generates purely interval food webs. Each species is randomly assigned three numbers: a niche value, a range radius proportional to the niche value and a range centre. Species feed on all species whose niche value falls into their range. The species with the smallest niche value has a range 0 to ensure the presence of, at least, one basal species. This defines the so-called adjacency matrix of the network: α with $\alpha_{ij} = 1$ if i preys upon j and $\alpha_{ij} = 0$ otherwise. The parameters of the probability distributions of the niche and the range are chosen in order to obtain an average connectance $C/2$ in the matrix α. For the construction of the corresponding interaction matrix, one multiplies the entries of α with positive i.i.d. values and the entries of α^t with negative i.i.d. values, and finally one adds up the two corresponding matrices. This results in a interaction matrix A with upper diagonal with mostly (but not exclusively) nonnegative entries, and lower diagonal entries mostly nonpositive. The average connectance of A is $C = L/S^2$. Notice that this construction is slightly different from the one proposed by Allesina and Tang in [15], since if species i preys on j and vice-versa, those interactions do not cancel out but are added in A. The combination of both interactions can either be mutualistic or competitive, or can remain a predator-prey interaction.

Nested-hierarchy model. Cattin et al. [28] proposed a model that tries to implicitly take evolution into account and relaxes the intervality of the diets of the niche model. The model creates a nested hierarchy between species driven by phylogenetic constraints. Two numbers

are randomly assigned to each species: a niche value and a number of prey that depends on the niche value. The species with the smallest niche value has no prey. After having reordered the species according to their niche value, prey are assigned to species starting with the species that has the smallest niche value, according to the following rule: for a consumer i, one chooses randomly a prey j with smaller niche value; then, one considers a pool of prey consisting of all prey consumed by other consumers of j; consumer i will then be assigned prey among this pool; if the pool is too small, choose another pool, and if this is still not possible, choose randomly a new prey. This algorithm builds the adjacency matrix of the network α, whose connectance is set on average to $C/2$ by tuning the parameters of the probability distribution of the niche values and the number of links of each species. The interaction matrix A is then constructed in the exact same way as for the niche model and the connectance is $C = L/S^2$.

S.2.3.3 Interaction strength

Intensity of interactions plays a key role for the local stability of networks [30, 31]. As already suggested by May's criterion [10], in the framework of the random model, interactions strength should decrease at least at rate $\frac{1}{\sqrt{CS}}$ in order to preserve stability when complexity CS increases. This consideration motivates the introduction of the normalising parameter $\delta \geqslant 0$ in (S2) for the study of ecological feasibility of equilibria. Three regimes naturally emerge: strong interactions ($\delta = 0$), moderate interactions ($\delta = 1/2$) and weak interactions ($\delta = 1$). A useful approach for the interpretation of these different regimes is the weight of a node in the network. Consider for example the random model. As the network is unstructured, all species have on average the same role, and the expected total weight of their interactions is

$$W_i = \frac{1}{(CS)^\delta} \sum_{j=1}^{S} \mathbb{E}\left(|a_{ij}|\right) = \frac{1}{(CS)^\delta} S\,\mathbb{E}\left(|a_{11}|\right) = (CS)^{1-\delta}\,\mathbb{E}\left(|a_{11}| \mid a_{11} \neq 0\right)$$

since a_{ij} are i.i.d. by assumption. One sees that for $\delta = 1$, W_i is constant and does not depend on the complexity. For $\delta = \frac{1}{2}$, the total weight is proportional to the square root of the complexity, and for $\delta = 0$, W_i depends linearly on the complexity. From the mathematical point of view, these three regimes correspond to three normalisation of the sum of i.i.d. random variables. Indeed, let $X_1, X_2, \ldots$ be a sequence of i.i.d. centred random variables. The rescaled sum $\frac{1}{n^\delta}\sum_{i=1}^n X_i$ converges almost surely to zero if $\frac{1}{2} < \delta \leqslant 1$ (law of large numbers). If $\delta = \frac{1}{2}$, the central limit theorem states that $\frac{1}{n^\delta}\sum_{i=1}^n X_i$ converges in distribution to a normally distributed random variable with mean zero and finite standard deviation. Proposition S.3.2 and Theorem S.3.5 below show the link between these probabilistic regimes and the nature of the equilibrium as the size of the network goes to infinity, depending on the parameter δ. While x^* converges to a deterministic value when interactions are weak ($\delta = 1$), it obeys a central limit theorem and converges to a well-defined random variable when the interactions are moderate ($\delta = \frac{1}{2}$). Finally, if $0 \leqslant \delta < \frac{1}{2}$, the rescaled sum does not converge and its standard deviation explodes. Consequently, the case of strong interactions ($\delta = 0$) is not studied, as the mathematical problem is asymptotically ill-posed.

S.2.4 Growth rates

The parameter r is of particular interest when studying feasibility as illustrated in [26]. For any fixed realisation of the random interaction matrix A, it is always possible to tune the parameter r in order to get any desired equilibrium. Choose any vector $u \in \mathbb{R}^S$ and set

$$r(u) = \left(-\theta I - \frac{A}{(CS)^\delta}\right) u$$

as a structural growth rates vector. A glance at (S3) shows immediately that the resulting equilibrium is $x^* = u$. Consider now the all-ones vector $\mathbb{1}$ in $\mathbb{R}^S$. This direction $\mathbb{1}$ is in a sense the most feasible, since it is as far as possible from the boundaries of the admissibility domain (i.e. the positive orthant). Setting the growth rates vector to the so-called structural vector $r(\mathbb{1})$ leads the equilibrium deterministically to $x^* = \mathbb{1}$. Interestingly, the authors of [20] studied stability using non-random interaction weights. They used $r(u)$ with $u = (-d/\theta)\mathbb{1}$, for some positive constant $d > 0$, to avoid transcritical bifurcations where the abundances of the steady state x_i^* become negative. They argue that with this particular choice, the only way in which a species can become extinct is via a degenerate bifurcation that makes a positive equilibrium unstable, causing the system to move suddenly to a different equilibrium point.

However, choosing the parameter r in such a way, i.e., contingently to A, is clearly uninformative as it erases the whole structure of the model, and nips the purpose of the randomisation in the bud. The parameter r should thus not be set according to a particular realisation of A. This can be achieved with, for example, the *mean structural vector* v defined by

$$v = \mathbb{E}\left(r(\mathbb{1})\right) = \mathbb{E}\left(-\theta I - \frac{A}{(CS)^\delta}\right) \cdot \mathbb{1}. \tag{S6}$$

This vector is deterministic, therefore clearly independent of the randomness of A, and it provides equilibria that are, on average, the most feasible. Choosing this v as growth rates vector allows then to study feasibility in the most favourable conditions. The mean structural vectors of the seven models of interest are given in Table 2.

The analytical results of Sections S.3.1.1 and S.3.2.1 hold for more general growth rates vector than v. They allow to consider random vectors $r = (r_i)$ with i.i.d. entries. In the case of unstructured models, v is indeed a particular case of this type of vectors, as the entries are all identical and deterministic, therefore independent of each other. In the following, we always illustrate our results with the mean structural vector v, unless specified.

S.3 Probability of feasibility

We study analytically the probability of feasibility P_S in the framework of the random model. For moderate interactions, P_S decreases exponentially with the size of the system. For weak interactions, P_S is asymptotically equal to one when the growth rate vector is set to v (see Eq. (S6)). We show that for other choices of growth vector, if the interactions are weak, then P_S either equals one or zero, depending on the value of the parameters. We provide estimations of P_S by means of Monte Carlo simulations for the other models and observe the same phenomenon. In the case of weak interactions, P_S can be estimated analytically for all unstructured models.

Model	Mean interaction	Mean structural vector
Random		$v_i = \lvert\theta\rvert - (CS)^{1-\delta}\,\mu_A$
Competition	$\mathbb{E}(a_{ij} \mid a_{ij} \neq 0) = \mu_A$	$v_i = \lvert\theta\rvert - \frac{C(S-1)}{(CS)^{\delta}}\,\mu_A$
Mutualism		$v_i = \lvert\theta\rvert - \frac{C(S-1)}{(CS)^{\delta}}\,\mu_A$
Predation		$v_i = \lvert\theta\rvert - (CS)^{1-\delta} \cdot \frac{\mu_{A_-}+\mu_{A_+}}{2}$
Cascade	$\mathbb{E}(a_{ij} \mid a_{ij} > 0) = \mu_{A_+} > 0$ and	$v_i = \lvert\theta\rvert - \frac{i-1}{(CS)^{\delta}}\,C\,\mu_{A_-} - \frac{S-i}{(CS)^{\delta}}\,C\,\mu_{A_+}$
Niche	$\mathbb{E}(a_{ji} \mid a_{ij} < 0) = \mu_{A_-} < 0$	*Simulated*
Nested hierarchy		*Simulated*

Supplementary Table B Models of interactions and their corresponding mean structural vector v. Note that the form of v is the same for the random, competition and mutualism models. In a competitive system, $\mu_A < 0$ and in a mutualistic one, $\mu_A > 0$. In all unstructured models (random, competition, mutualism, predation), the mean structural vector has the same direction as the all-ones vector, meaning that the structural growth rates are the same for all species. For the structured models (cascade, niche and nested hierarchy), species do have different roles in the network, while in the other models all species behave, on average, identically. The mean structural vector follows then a particular direction which is no more the all-ones vector. One sees that, for the cascade model, for appropriate choice of the parameters θ, μ_{A_+} and μ_{A_-} leads to a vector v which has negative entries for species with small indices i (top predators) and positive entries for species with large indices (basal species). This type of growth rate vector is a characteristic of such networks [69, 70]. For the niche and the nested hierarchy models, the algorithmic construction of the interaction matrix makes the formula of their respective v very complicated. They are therefore obtained by Monte Carlo simulations. As for the cascade model, negative growth rates are observed.

S.3.1 Moderate interactions

We show that in the random model with moderate interactions, there exists asymptotically almost surely no feasible equilibrium of the system (S1). The lack of structure of this model brings independence of the entries of x^*, the biomasses at equilibrium. Moreover, the rate of normalisation $\delta = \frac{1}{2}$ makes these entries follow asymptotically a normal law. Thus, when the number of species becomes large, there is a smaller and smaller probability that all of them have positive equilibrium, as illustrated in Suppl. Fig. A and Fig. 1, Main Text.

This phenomenon is a key feature of this model and does not depend on the choice of the intrinsic rates r. Indeed, we can show analytically that the probability of persistence at equilibrium goes towards zero for any choice of a random vector r with i.i.d. entries. This framework contains the particular case where the intrinsic rates are all equal, i.e. the same direction as v, the mean structural vector of the random model.

We extend this study by estimating P_S in the other unstructured and structured models by simulations. For each model, one samples independent realisations of the interaction matrix A and sets the growth rates vector to the mean structural vector v of the model. The equilibrium is found according to Equation (S3) and P_S is estimated by the proportion at which the equilibrium is admissible. The simulation results are analogous to the random model in the sense that P_S decreases exponentially towards zero with S. Note that we also performed simulations for the random model, whose results correspond nicely to the analytical prediction (see Suppl. Fig. A (a)).

S.3.1.1 Analytical results

First, we express the law of x_i^* for arbitrary large S. Our results ensue from the direct application, with some extensions, of Geman's work [33] on solution of random large systems. They hold under the following assumptions:

Assumption S.3.1. In the Lotka-Volterra model (S1), we assume that

(i) $\delta = \frac{1}{2}$;

(ii) the interaction matrix $A = (a_{ij})$ has i.i.d. entries with common mean $\mathbb{E}(a_{11}) = 0$;

(iii) the intrinsic growth rates vector $r = (r_i)$ has i.i.d. entries;

(iv) r and A are independent;

(v) the second moments of the laws of A and r satisfy $\frac{\mathbb{E}(r_1^2)\mathbb{E}(a_{11}^2)}{|\theta|^2} < \frac{1}{4}$;

(vi) there exists a constant κ such that $\mathbb{E}(|a_{11}|^S) < S^{\kappa S}$, for all $S \geq 2$;

(vii) the matrix $(\theta I + A/\sqrt{CS})$, where I denotes the $S \times S$ identity matrix, is nonsingular.

The assumptions (v) and (vi) on the second and higher moments of A and r are very natural and not restrictive, since they are satisfied by a wide collection of laws (normal, uniform, beta, gamma, lognormal,...) as long as the chosen standard deviation is not too large. Note also that the mean structural vector satisfies these assumptions. The last condition allows us to consider that the solution to (S3) always exists.

Proposition S.3.2. *Under Assumptions S.3.1, the biomasses at equilibrium of the model* (S1) *converge in law towards Gaussian random variables:*

$$x_i^* \overset{\mathcal{L}}{\Rightarrow} \mathcal{N}\left(-\frac{\mathbb{E}\left(r_1\right)}{\theta},\ \frac{\mathrm{Var}(r_1)}{\theta^2}+\frac{\mathbb{E}\left(r_1^2\right)\sigma^2}{\theta^2\left(\theta^2-\sigma^2\right)}\right), \quad \textit{for all } i=1,\ldots,S. \tag{S7}$$

Moreover, for every fixed $1\leqslant k\leqslant S$, *the collection* $(x_1^*,...,x_k^*)$ *has independent entries.*

Proof. The proof is a generalisation of a result given in [33]. We will assume without loss of generality that $C=1$. Indeed, $\mathbb{E}(\frac{1}{\sqrt{C}}a_{ij})=0$ and $\mathrm{Var}(\frac{1}{\sqrt{C}}a_{ij})=\mathrm{Var}(a_{ij}|a_{ij}\neq 0)$ so that in the case of moderate interactions the variance of the a_{ij} is preserved when dividing any a_{ij} by $\sqrt{C}$. Consider the system (1),

$$-\theta x^* = r+\frac{1}{\sqrt{S}}Ax^*.$$

First approach the solution of the system by a Neumann progression which is given by

$$-\theta x^* = r+\sum_{k=1}^{\infty}\left(\frac{A}{\sqrt{S}}\right)^k\left(-\frac{1}{\theta}\right)^k r.$$

As in [33], define

$$\alpha_{r,\theta}\left(i,k,S\right)=\left[\left(\frac{A}{\sqrt{S}}\right)^k\left(-\frac{1}{\theta}\right)^k r\right]_i=\left(-\frac{1}{\theta}\right)^k\left(\frac{1}{\sqrt{S}}\right)^k\sum_{l_1,\ldots,l_k}a_{il_1}a_{l_1l_2}...a_{l_{k-1}l_k}r_{l_k}, \tag{S8}$$

which is simply the ith component of $\left(\frac{A}{\sqrt{S}}\right)^k\left(-\frac{1}{\theta}\right)^k r$. We compute the joint moments of the $\alpha_{r,\theta}$ and show that they are the same as those of normal random variables. For m fixed and distinct pairs $(k_j,n_j)_{1\leqslant j\leqslant m}$ we thus compute $\mathbb{E}\left(\prod_{j=1}^m \alpha_{r,\theta}\left(i_j,k_j,S\right)^{n_j}\right)$. This involves the same combinatorics as the one employed in [33] as mixing terms from (S8) arise. Indeed for asymptotical contributions, each chain $a_{l_1l_2}...a_{l_{k-1}l_k}r_{l_k}$ has to be paired exactly with itself. Using moreover independence of the (a_{ij}) and the (r_i), this gives the joint moments

$$E\left(\prod_{j=1}^m\alpha_{r,\theta}\left(i_j,k_j,S\right)^{n_j}\right)=\begin{cases}\prod_{j=1}^m\mathbb{E}(r_1^2)^{\frac{n_j}{2}}\left(\frac{\sigma}{\theta}\right)^{k_jn_j}\prod_{p=1}^{n_j/2}(2p-1) & \text{if every } n_j \text{ are even}\\ 0 & \text{otherwise.}\end{cases}$$

These are the joint moments of independent Gaussian random variables, i.e.

$$\left(\alpha_{r,\theta}\left(i_1,k_1,S\right),...,\alpha_{r,\theta}\left(i_m,k_m,S\right)\right)\overset{\mathcal{L}}{\Rightarrow}\left(Z_1,...,Z_m\right),$$

where $Z_j\sim\mathcal{S}\left(0,\ \mathbb{E}\left(r_1^2\right)\frac{\sigma^{2k_j}}{\theta^{2k_j}}\right)$ are independent. Returning now to the Neumann progression and by calculating variance and expectation, we find that when $S\to\infty$

$$\lim_{S\to\infty}x_i^*=x_i^{*,\infty}\sim\mathcal{N}\left(-\frac{\mathbb{E}\left(r_1\right)}{\theta},\ \frac{\mathrm{Var}(r_1)}{\theta^2}+\frac{\mathbb{E}\left(r_1^2\right)\sigma^2}{\theta^2\left(\theta^2-\sigma^2\right)}\right).$$

■

This result is similar to that obtained in [71, equations (16.12) and (16.21)], where mean, variance, and coefficient of variation have been computed. The normal asymptotical distribution and the fact that the equilibria are asymptotically i.i.d. from the previous Proposition S.3.2 allow to go one step further and to show that there exists almost surely no feasible equilibrium for species-rich systems of type (1).

Theorem S.3.3. *Under Assumption S.3.1, the probability that an equilibrium of model* (S1) *is feasible tends toward zero. That is* $\lim_{S\to\infty} P_S = 0$.

Proof. Let us define $P_S^{(k)} = \mathbb{P}\left(x_j^* > 0, \forall j = 1, \dots, k\right)$. With this notation, $P_S = P_S^{(S)}$. The convergence in law in (S7), as well as the independence of (x_i^*) imply

$$\begin{aligned} 0 \leqslant \lim_{S\to\infty} P_S &\leqslant \limsup_{S\to\infty} P_S^{(S)} \\ &\leqslant \limsup_{S\to\infty} P_S^{(k)} \\ &= \mathbb{P}\left(x_j^* > 0, \forall j = 1, \dots, k\right) \\ &= \Phi\left(\frac{\mathbb{E}(r_1)}{\sqrt{\mathrm{Var}(r_1) + \mathbb{E}\left(r_1^2\right)\frac{\sigma^2}{\theta^2-\sigma^2}}}\right)^k, \end{aligned}$$

for all $k \geqslant 1$ and where Φ denotes the standard Gaussian cumulative distribution function. Since the probability on the right-hand side is strictly less than 1, this implies that $\limsup_{S\to\infty} P_S = 0$. ■

When considering the mean structural vector of the random model $r = v$ (see Suppl.Table 2), the previous proof allows to approximate the probability that an equilibrium is feasible in the following way:

$$P_S \approx \Phi\left(\sqrt{\frac{\theta^2-\sigma^2}{\sigma^2}}\right)^S, \tag{S9}$$

which is represented in Suppl. Fig. A.

S.3.1.2 Simulations

The exponential decrease of P_S is not restricted to the random model, for which our analytical result holds. Indeed, we have computed numerically the probability of feasibility for the other models, as well as for the random model (see Fig. 1, Main Text). The growth rates vector is set to the mean structural vector for each model respectively (Table 2).

An interaction is non-zero with probability C. We choose $a_{ij} \sim \mathcal{N}(0,\sigma)$ for any non-zero entries of the interaction matrix A in the random model, i.e. when the sign of the interaction does not matter. In this sense, $\mathbb{E}(a_{ij}) = 0$ and $\mathrm{Var}(a_{ij}) = C \cdot \mathrm{Var}(a_{ij}|a_{ij} \neq 0) = C \cdot \sigma^2$. In the other cases, a strictly positive interaction is randomly drawn from a folded normal distribution such that $a_{ij} \sim |\mathcal{N}(0,\sigma)|$. The expectation is $\mathbb{E}(a_{ij}|a_{ij} \neq 0) = \sigma\sqrt{2/\pi}$ and the variance $\mathrm{Var}(a_{ij}|a_{ij} \neq 0) = \sigma^2(1-2/\pi)$. A strictly negative interaction is similarly sampled such that $a_{ij} \sim -|\mathcal{N}(0,\sigma)|$.

In Fig. 1 of Main Text, the connectance as been fixed to $C = 0.25$ for each model. To reach such a connectance in the case of the niche model, we choose the niche values uniformly on the interval $[0; 1]$, and their breadth according to a Beta random variable with shape parameters $(1, 3)$.

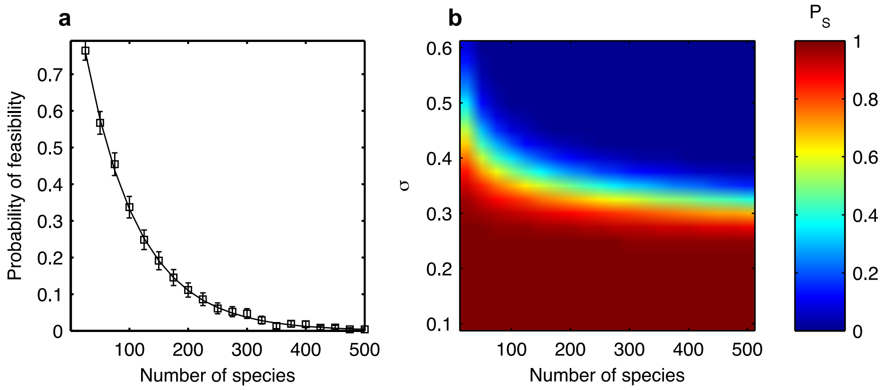

Supplementary Figure A. Probability of feasibility as a function of number of species S and standard deviation of interaction strengths σ in the random model under moderate interactions and with the mean structural vector. (**a**) The continuous line denotes the analytical predictions of P_S, while the error bars are 95% confidence intervals from Monte-Carlo estimates for 1000 simulations. The parameters are $C = 1$, $\sigma = 0.4$, $\theta = -1$. (**b**) Analytical prediction of the probability of feasibility with respect to σ and S.

In the case of the niche and nested-hierarchy models, we also simulated the equilibria feasibility when not using the mean structural vector v. In Suppl. Fig. B, the growth rates are i.i.d. gaussian random variables with standard deviation fixed to 0.15 and mean one (in Suppl. Fig. B (a-d)) or mean zero (in Suppl. Fig. B (e-f)). In the former, the probability of feasibility rapidly decreases towards zero, similarly as what is predicted with random models. When $\mathbb{E}(r_i) = 0$ for all i, the equilibria abundances are centered around zero with a positive variance independently of the species index i. As such, there is no chance to observe $P_S > 0$ for this choice of r_i.

S.3.2 Weak interactions

For the case $\delta = 1$, we provide the conditions under which the system (S1) possesses almost surely a feasible equilibrium. In this situation, the interactions between the species become extremely weak when the size of the system grows. Compared to the moderate case, the variance of the solution approaches zero allowing us to use a law of large numbers. This drives each component x_i^* of the solution to a constant proportional to its intrinsic growth rate r_i as illustrated in Suppl. Fig. C. This leads to feasible equilibria for any positive growth rate.

Analytical results are given in the case of unstructured models, whereas structured models are explored by mean of simulations.

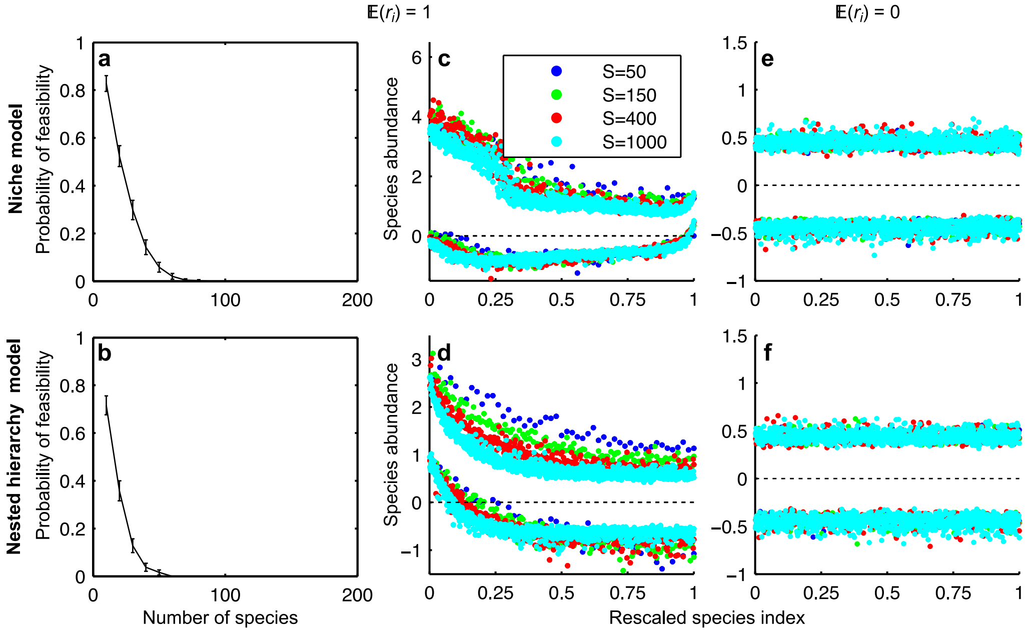

Supplementary Figure B. Equilibria simulated from the niche and nested-hierarchy models under moderate interactions and for i.i.d. random growth rates. (**a-b**) The probability of feasibility decreases rapidly when $E(r_i) = 1$. Envelop (the maximum and the minimum value) of the equilibria for (**c-d**) $\mathbb{E}(r_i) = 1$ and (**e-f**) $\mathbb{E}(r_i) = 0$. In this case P_S is always zero. The species have been assigned a number between zero (top species) and one (basal species) corresponding to their hierarchy in the web. 500 simulations have been performed for each S. The growth rates are normally distributed with a fixed standard deviation $\sqrt{\mathrm{Var}(r_i)} = 0.15$.

S.3.2.1 Analytical results

We begin by giving the assumptions under which our analytical results hold :

Assumption S.3.4. In the Lotka-Volterra model (S1), we assume that

(i) $\delta = 1$;

(ii) the interaction matrix $A = (a_{ij})$ has i.i.d. entries with common mean $\mathbb{E}(a_{11}) = C\mu_A$ so that $|\mu_A| < |\theta|$;

(iii) the intrinsic growth rates vector $r = (r_i)$ has bounded entries;

(iv) the law of the (a_{ij}) satisfies $\mathbb{E}(a_{ij}^8) < \infty$;

(v) the matrix $(\theta I + A/(CS))$ is nonsingular.

As for the Assumptions S.3.1, these conditions are not restrictive on the choice of the distributions for the (a_{ij}). The mean structural vector satisfies the Assumption S.3.4 (iii) for all the models that are considered. In (ii), $\mathbb{E}(a_{11}) = C\mu_A$ is equivalent to $\mathbb{E}(a_{11} \mid a_{11} \neq 0) = \mu_A$

since C is the probability that an entry of the matrix is set to 0. Note furthermore that, in the settings of weak interactions, the entries of A are divided by CS so that $\mathbb{E}(a_{ij}/(CS)) = \mu_A/S$.

We first introduce the analytical result for the random model.

Theorem S.3.5. *Under Assumption S.3.4 with $\mu_A = 0$ the asymptotic equilibrium of the model* (S1) *is feasible and is given by*

$$x_i^{*,\infty} = \lim_{S\to\infty} x_i^* = \frac{r_i}{|\theta|} \text{ almost surely,} \qquad \text{for all } i = 1, \dots, S.$$

Moreover, $x_i^{*,\infty}$ is almost surely feasible for any r such that $r_i > 0$ for all i.

Proof. The convergence of x_i^* toward $\frac{r_i}{|\theta|}$ follows directly from results on the solution of large random systems of linear equations, [33, Thm. 1]. ■

In the case of the random model, setting the growth rates vector to $v = |\theta| \cdot \mathbb{1}$ leads thus to $x_i^{*,\infty} = 1$ for all i. But Theorem S.3.5 is more general and allows to consider any growth rates vector r with bounded entries. In Suppl. Fig. C for example, the entries of r are chosen to take only two values and one sees that the equilibrium vector also converges only toward two values (proportional to r_i) when S becomes large.

In Assumption S.3.4, the expectation μ_A does not need to be zero. This enables the derivation of analytical results in the case of random models in which the mean interaction is positive (mutualistic and commensalistic interactions) or negative (competitive and amensalistic interactions). Indeed, a generalisation of the previous proof by allowing arbitrary μ_A leads to the following:

Theorem S.3.6. *Under Assumption S.3.4, the asymptotic equilibrium of the model* (S1) *is given by*

$$x_i^{*,\infty} = \lim_{S\to\infty} x_i^* = \frac{r_i}{|\theta|} + \frac{\mu_A}{|\theta|\left(|\theta| - \mu_A\right)}\bar{r} \qquad \text{almost surely, for all } i = 1, \dots, S,$$

where $\bar{r}$ denotes the arithmetic mean of the entries of r. Moreover, if $r_i \geqslant \frac{\mu_A}{\mu_A+\theta} \cdot \bar{r}$ for all $i = 1, \dots, S$, then $x^{*,\infty}$ is feasible with probability one.

Proof. The proof is analog to the one provided in [33, Thm. 2], where we write $\hat{W} = W - M$ with M the $S \times S$ matrix with every component set to $C\mu_A$, $W = A$ and $\hat{W}$ is an $S \times S$ random matrix with i.i.d. centred entries. ■

However, the competition and mutualistic models introduced in Section S.2.3.1 do not completely satisfy the Assumptions S.3.4, since some dependences are introduced among the entries of the interaction matrix A. Indeed, $a_{ij} = 0 \Leftrightarrow a_{ji} = 0$, so that commensalistic/amensalistic interactions are forbidden. Moreover, the particular case of predation leads to a sign antisymmetric matrix A, i.e. $a_{ij} > 0 \Leftrightarrow a_{ji} < 0$. In the following, we show that the same type of results are obtained for these cases.

For mutualism or competition, we define the entries of A in the following way,

$$a_{ij} = w_{ij} \cdot b_{ij} \quad \text{and} \quad a_{ji} = w_{ji} \cdot b_{ij},$$

where (w_{ij}) are i.i.d. random variables of mean μ_A and variance σ^2 (e.g. folded normal random variables) and (b_{ij}) are i.i.d. Bernoulli random variables of parameter C.

Note that for every $i, j \in \mathcal{S}$, $\mathbb{E}(a_{ij}) = \mathbb{E}(a_{ji}) = C\mu_A$, so that if we define M to be the matrix with all elements set to $C\mu_A$, the matrix

$$\hat{W} = A - M \tag{S10}$$

is centred and that $Cov(\hat{w}_{ij}, \hat{w}_{ji}) = \mu_A^2 \cdot C(1-C) \neq 0$. Like in the proof [33, Thm. 2], we need to show that $\|\hat{W}/S\| \to 0$. This assertion is based on [72], which gives an upper bound for the norm of sample covariance matrix, and can be related to the work in [73], where the largest eigenvalue of random symmetric matrices is studied. Here we will show that $\|\hat{W}/S\| \to 0$ in the framework of [74], where it is demonstrated that the limiting distribution of the eigenvalues of a sample covariance matrix $1/SVV^T$ remains the Marčenko-Pastur law, even with some dependencies among the entries of a centred random matrix V.

Lemma S.3.7. *Consider the random matrix* $\hat{W}$ *defined by equation* (S10) *and assume that there exists a constant* K *such that* $\mathbb{E}(|\hat{w}_{ij}|^k) \leqslant K^{2k}$ *for any* $k \leqslant S$ *and* $1 \leqslant i, j \leqslant S$*. Then*

$$\|\hat{W}/S\| \to 0 \quad \textit{almost surely.}$$

Proof. The conditions (MP1), (MP2) and (MP3) in [74] still hold for $\hat{W}$ in our framework. Thus letting λ_{max} be the largest eigenvalue of $1/S\hat{W}\hat{W}^T$, the same combinatorial arguments can be used, and following [74, Section 4] we arrive to

$$\begin{aligned}\mathbb{E}(\lambda_{max}^k) \leqslant \mathbb{E}\left(\mathrm{Tr}(\frac{1}{S}\hat{W}\hat{W}^T)^k\right) &\leqslant \frac{(2k)!}{k!(k+1)!}S + c \cdot K^{2k} + \mathcal{O}(1/S) \\ &< 4^k S + c \cdot K^{2k} + \mathcal{O}(1/S),\end{aligned}$$

where c is a constant and $\mathrm{Tr}(\cdot)$ the trace operator. Letting $\epsilon > 0$ and using the Markov inequality, we find

$$\mathbb{P}\left(\frac{1}{\sqrt{S}}\left\|\frac{1}{\sqrt{S}}\hat{W}\right\| > \epsilon\right) = \mathbb{P}\left(\lambda_{max}^k > S^k\epsilon^{2k}\right) \leqslant \frac{4^k S + cK^{2k}}{S^k\epsilon^{2k}} + \mathcal{O}(1/S^{k+1}),$$

which goes to zero when S is large and since $\sum_S \mathbb{P}\left(\frac{1}{\sqrt{S}}\left\|\frac{1}{\sqrt{S}}\hat{W}\right\| > \epsilon\right) < \infty$ for $k \geqslant 3$, almost sure convergence holds. ■

Corollary S.3.8. *Under Assumption S.3.4 with a matrix* A *so that* $a_{ij} = 0 \Leftrightarrow a_{ji} = 0$*, and where* $\mathbb{E}(a_{ij}|a_{ij} \neq 0) = \mu_A$ *and* $r = \gamma \cdot \mathbb{1}$*, with* $\gamma \in \mathbb{R}^*$*, the asymptotic equilibrium of the model* (S1) *is given by*

$$x_i^{*,\infty} = \lim_{S\to\infty} x_i^* = \frac{\gamma}{|\theta| - \mu_A} \quad \textit{almost surely,}$$

for all $i = 1, \dots, S$*. Under these assumptions and for* $\gamma/(|\theta| - \mu_A) > 0$*,* $x^{*,\infty}$ *is feasible with probability one.*

Proof. Since each line in A, i.e. the collection $(a_{ij})_{1\leqslant j\leqslant S}$ for arbitrary $i \in \mathcal{S}$, still contains independent elements for arbitrary S, the proof is analog to the one of Thm S.3.6 by using Lemma S.3.7. ∎

In the case of predation, interactions have the form $(+,-)$, so that the previous Corollary extends in the following way.

Corollary S.3.9. *Consider Assumption S.3.4 with a matrix A so that $\mu_A = \frac{\mu_{A_+}+\mu_{A_-}}{2}$, where $\mathbb{E}(a_{ij}|a_{ij} > 0) = \mu_{A+} > 0$, $\mathbb{E}(a_{ij}|a_{ij} < 0) = \mu_{A_-} < 0$, and with $a_{ij} = 0 \Leftrightarrow a_{ji} = 0$ and $\mathrm{sign}(a_{ij}) = -\mathrm{sign}(a_{ji})$. Then for $r = \gamma \cdot \mathbb{1}$, with $\gamma \in \mathbb{R}^*$, the asymptotic equilibrium of the model* (S1) *is given by*

$$x_i^{*,\infty} = \lim_{S\to\infty} x_i^* = \frac{\gamma}{|\theta| - \frac{\mu_{A_+}+\mu_{A_-}}{2}} \text{ almost surely,}$$

for all $i = 1, \ldots, S$. Under these assumptions and for $\gamma / \left(|\theta| - \frac{\mu_{A_+}+\mu_{A_-}}{2}\right) > 0$, $x^{*,\infty}$ is feasible with probability one.

Consequently, the mean structural vector (see Suppl. Table 2) leads almost surely x^* to the vector $\mathbb{1}$ for every unstructured model.

S.3.2.2 Simulations

Unstructured networks. As in the moderate case, simulations have been performed to illustrate our analytical results. We illustrate the results for the random model in Suppl. Fig. C. The outcomes for the other unstructured models are analogous. All random variables a_{ij} are defined as in Section S.3.1.2, with $C = 0.25$, $\sigma = 0.4$ and $\theta = -1$.

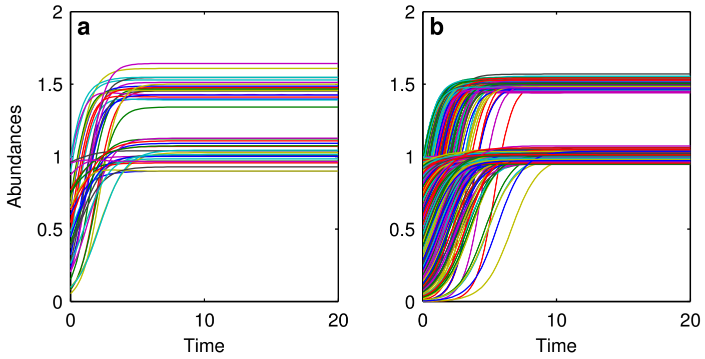

Supplementary Figure C. Trajectories of the abundances of species through time in the case of weak interactions ($\delta = 1$) for the random model. Half of the species have a growth rate set to 1, the other half set to 1.5. As predicted analytically (Thm S.3.5), the abundances stabilise around their intrinsic growth rates ($|\theta| = 1$ and $\sigma = 0.4$). The simulations have been performed with MATLAB ode45. (**a**) With 36 species. (**b**) With 500 species. Note that for $S \to \infty$, equilibrium abundances converge to 1 and 1.5.

Structured networks. The case of the cascade model [17] is illustrated in Suppl. Fig. D (a) and (b). We represent the envelop of the equilibria for different S from 1000 simulations with $C = 0.25$, $\sigma = 0.4$ and $\theta = -1$. The standard deviation of any x_i^* decreases towards zero when S increases, independently of the role of species i. Using the mean structural vector, the same convergence to the equilibrium $\mathbb{1}$ as in the random model is hence obtained. We hypothesise that this result is a consequence of the cascade model yielding adjacency matrices with inferior triangular parts constructed like a Erdős-Rényi network. The distribution of a top, an intermediate and a basal species are illustrated in Suppl. Fig. E (a) and shows that any species abundance is independent of its role in the web, exactly as in random models.

For the niche model [18] and the nested-hierarchy model [28], a particular phenomenon occurs that is not observed for the other models. Indeed, simulations show that the equilibrium remains random when S increases. This result is likely attributable to the construction of the models, with the standard deviation of the abundances depending on the hierarchical position of the species. Importantly, standard deviations do not decrease towards zero as the size of the network grows, as illustrated in Suppl. Fig. D (d) and (f). Indeed, these highly structured networks and their related mean structural growth rates induce correlations among the abundances at equilibrium that prevent a convergence to a deterministic value, but rather takes place on a compact support. This is illustrated by the envelop of the equilibria that is represented in Suppl. Fig. D (c) and (e) for different values of S. The empirical distribution of x_i^* for particular species roles i is illustrated in Suppl. Fig. E. From these results, it is apparent that basal species may rapidly converge to a deterministic constant. However, for intermediate and top species, simulations show that the convergence is very likely to occur on a non-trivial support.

We also tested i.i.d. Gaussian random growth rates with $\sigma_r = \sqrt{\mathrm{Var}(r_i)} = 0.15$ for the niche and the nested-hierarchy models in Suppl. Fig. F. When $\mathbb{E}(r_i) = 1$, a similar phenomenon as what was observed with the mean structural vector appears. The standard deviations of x_i^* converge towards positive numbers and the resulting distribution of x_i^* depends on the species index i. However, when $\mathbb{E}(r_i) = 0$ or when the standard deviation of r_i increases, this particular hierarchical behavior disappears, as illustrated in Suppl. Figs. F and G. Every equilibria x_i^* are similarly distributed around zero.

S.3.3 Empirical networks

We used our method on empirical food webs to illustrate how species abundances at equilibrium depend on their intrinsic role in the web. The topology is thus fixed to the observed one and the interactions are modeled as for any random structured web (see the section Method in the Main Text). We report the results of four arbitrary networks. In Suppl. Fig. H, the mean structural vector is used. In Suppl. Fig. I two different random vectors with i.i.d. components are chosen. The distributions under weak interactions are also illustrated in Suppl. Fig. E, to allow better comparison with the niche and the nested-hierarchy models.

S.3.4 Connectance and interactions strengths

In our approach, we fixed the connectance C to arbitrary values in the mathematical developments, and to fixed values in the simulations. In this section, we briefly explore the consequence

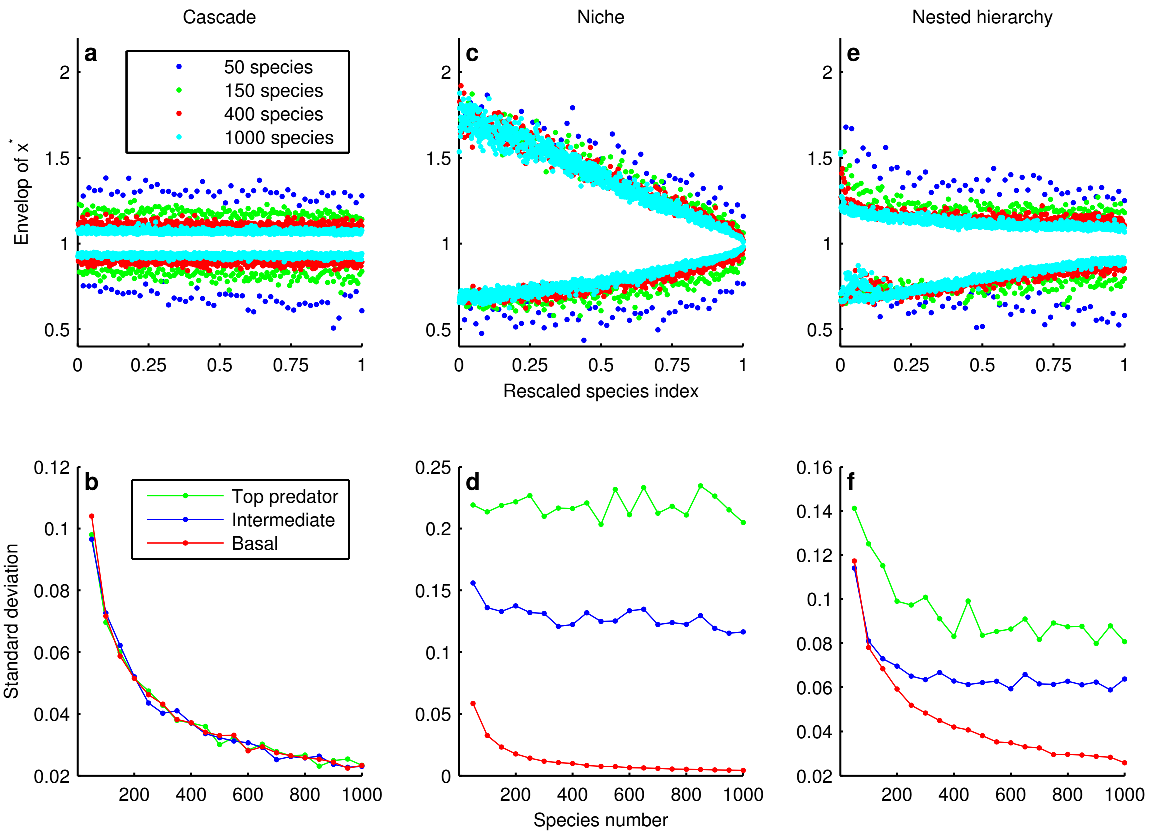

Supplementary Figure D. Behaviour of the structured models under weak interactions with the mean structural vector. In the first row, we plot for different values of S the envelop (the maximum and the minimal value) of the equilibrium x^* among 1000 simulations. The species have been assigned a number between zero (top species) and one (basal species) corresponding to their hierarchy in the web. In the second row, the standard deviations of x_i^* for $i = 1$ (top predator), $i = S/2$ (intermediate) and $i = S$ (basal) are represented as a function of S. The parameters are $C = 0.25$, $\sigma = 0.4$ and $\theta = -1$. (**a** and **b**) In the cascade model, although hierarchically ordered, species tend to behave similarly with regard to convergence as S grows. The equilibrium converges almost surely to the vector $\mathbb{1}$. (**c** - **f**) The niche and the nested-hierarchy models keep the randomness of the equilibrium when S grows, even under weak interactions.

of a relationship between connectance and species number on feasibility, as observed in several contributions, e.g. [75, 42]. Observe first that, from the point of view of the average interaction strength, introducing zeros with a given probability $1 - C$ in the interaction matrix is the same as multiplying the whole matrix by C. Therefore, one has

$$\frac{A}{(CS)^\delta} = \frac{BC}{(CS)^\delta},$$

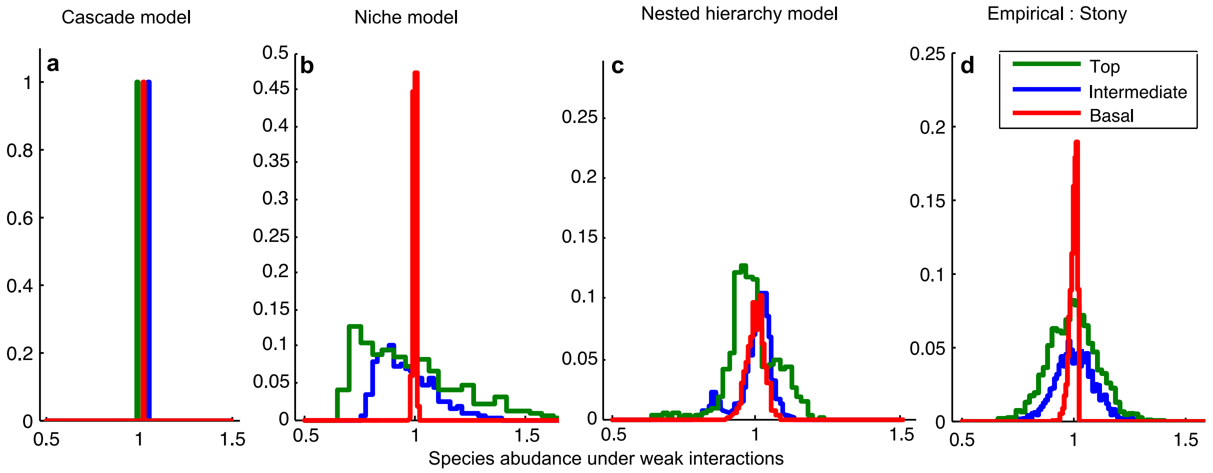

Supplementary Figure E. Empirical distribution of top, intermediate and basal species under a regime of weak interactions. (**a**) Cascade model with $S = 800$. (**b**) Niche model with $S = 800$. (**c**) Nested-hierarchy model with $S = 800$. The same parameters as in Suppl. Fig. D have been used among 1000 simulations. (**d**) The distribution of a top, intermediate and basal species from the empirical food web Stony is illustrated. The interactions strength have been simulated similarly as for any structured web (see Section Method in Main Text). The mean structural vector has been simulated with Monte-Carlo methods (200 trials).

where B is a matrix with all entries i.i.d. and such that $\mathbb{P}(b_{ij} = 0) = 0$. Consider $C = \frac{1}{S^\beta}$, for $\beta \geqslant 0$, which is a flexible function that adequately captures observed relationships between C and S. We get

$$\frac{BC}{(CS)^\delta} = \frac{B}{S^{\delta+\beta(1-\delta)}}.$$

In the Main Text and above, we showed that feasibility is warranted when $\delta + \beta(1-\delta) > \frac{1}{2}$, i.e. for

$$\beta > \frac{(\frac{1}{2} - \delta)}{(1-\delta)}.$$

Taking $\beta \geqslant \frac{1}{2}$ leads almost surely to feasible equilibria in the models considered here, independently of the exponent δ. For $\beta < \frac{1}{2}$, δ can be smaller than 0.5. This shifts the three previously described regimes for δ to the left, so that δ can be smaller to reach the same results. Consequently, the existence of a relationship between C and S does not affect our conclusions.

S.4 Consequences on local stability analysis

Any local stability analysis should be preceded by a feasibility analysis [27, 22, 76]. Indeed, studying the local stability of a point that is not biologically feasible is not instructive on the behaviour of the network dynamics. Moreover, the Jacobian matrix of the system (S4), therefore its eigenvalues too, depend explicitly on x^*. Therefore, there is no warranty that randomly

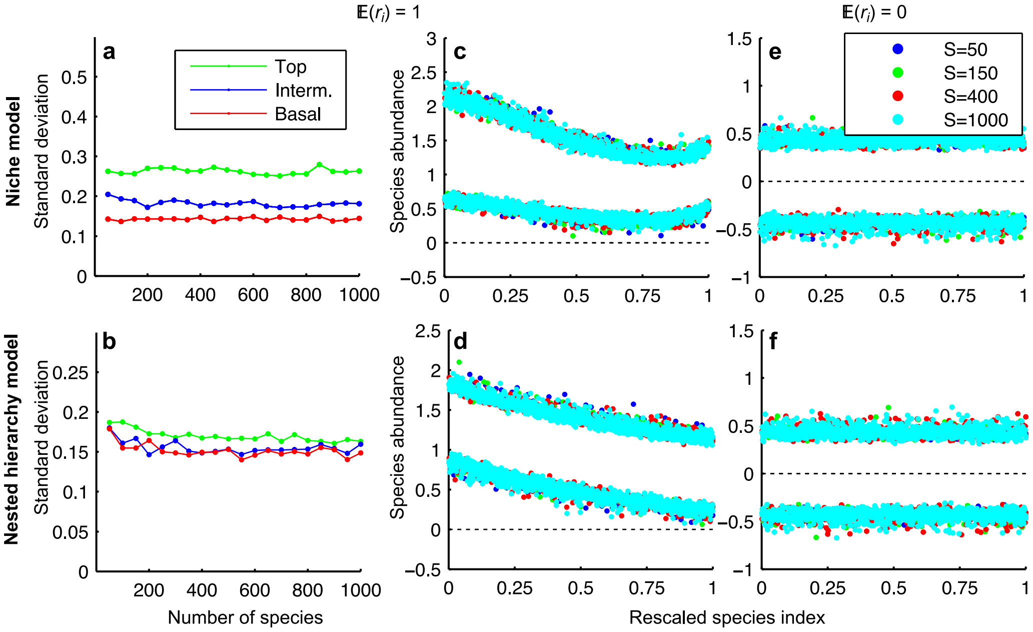

Supplementary Figure F. Equilibria simulated from the niche and nested-hierarchy models under weak interactions and for i.i.d. random growth rates. (**a-b**) The standard deviations of a top, an intermediate and a basal species are illustrated as a function of S for $E(r_i) = 1$. Envelop of the equilibria for (**c-d**) $\mathbb{E}(r_i) = 1$ and (**e-f**) $\mathbb{E}(r_i) = 0$. 500 simulations have been performed for each S. The growth rates are normally distributed with a fixed standard deviation $\sigma_r = 0.15$.

sampling directly the Jacobian may be sufficient to address local stability. Here, we explore this question, which extends the analyses of May and Allesina and co-workers [10, 15].

S.4.1 Random networks

We first focus on the random model of May [10] in the case of moderate interactions in the Proposition S.4.1 below. In this case, we find that May's criterion still holds when we randomly sample the interaction matrix A (and not directly the Jacobian) under the additional condition that the equilibrium x^* is feasible.

S.4.1.1 Moderate interactions

In Proposition S.4.1, we first show that May's criterion for stability, i.e.

$$\sqrt{CS}\tilde{\sigma} < |\theta|,$$

is still sufficient for any feasible equilibrium when $\tilde{\sigma}$ is the standard deviation of the normalised interaction matrix $A/(CS)^\delta$, and not the standard deviation of the Jacobian matrix of the system. Recall that the case of moderate interactions is here very natural, as relying on Wigner's [12] and Girko's [77] original convergence results. The criterion simply becomes

$$\sigma < |\theta|,$$

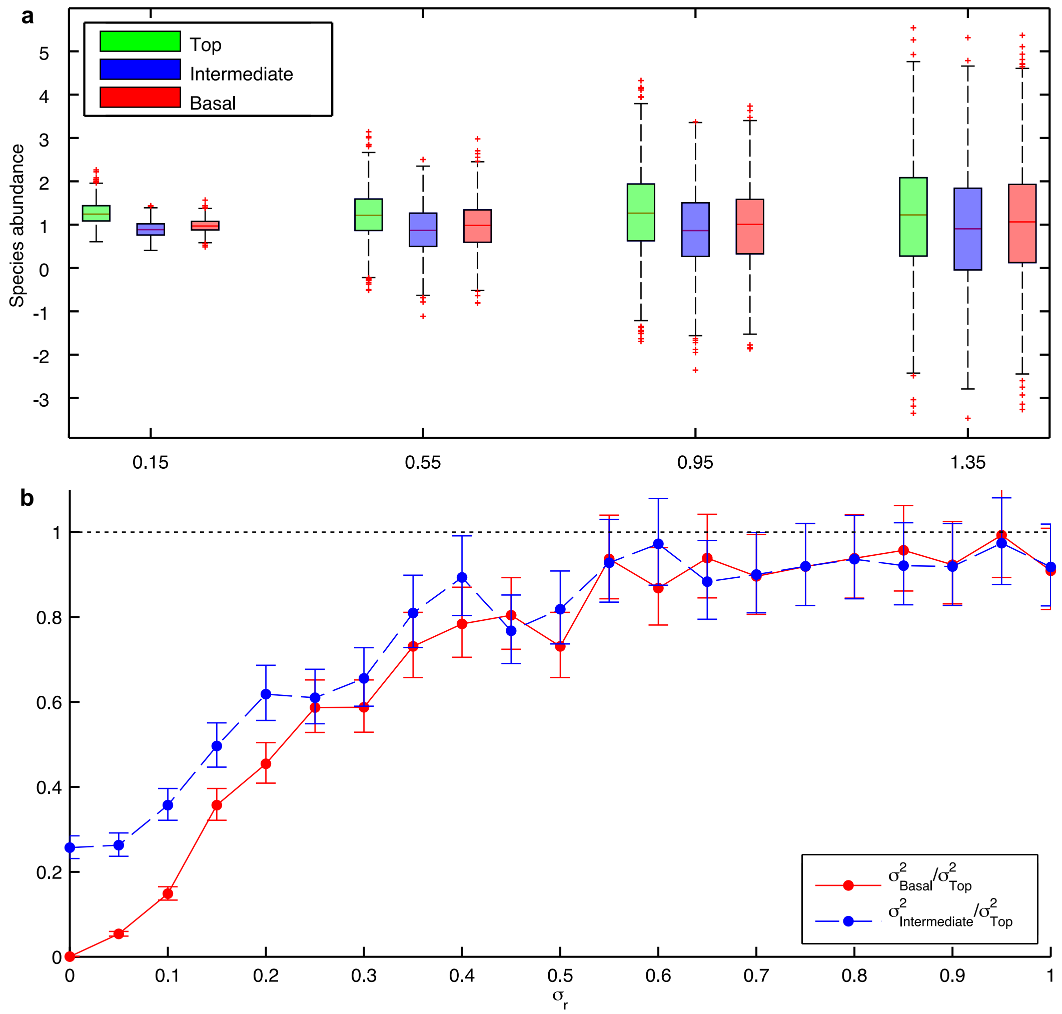

Supplementary Figure G. When the standard deviation σ_r of i.i.d. random growth rates increases, equilibria variances of top, intermediate and basal species tend to become similar under weak interactions in a structured food-web (illustration for the niche model). (**a**) Boxplot representation of the distributions. (**b**) Illustration of the variances ratio between basal and top (red trace, $\sigma^2_{\text{Basal}}/\sigma^2_{\text{Top}}$) and intermediate and top (blue trace, $\sigma^2_{\text{Intermediate}}/\sigma^2_{\text{Top}}$) as a function of σ_r with a 95% confidence interval. 1000 simulations have been performed for each σ_r, $S = 800$ and the growth rates are normally distributed with $E(r_i) = 1$.

where σ is the standard deviation of A.

Proposition S.4.1. *Under Assumption S.3.1 and if*

$$\sigma < |\theta|,$$

any asymptotic feasible equilibrium of the model (S1) *is almost surely linearly stable.*

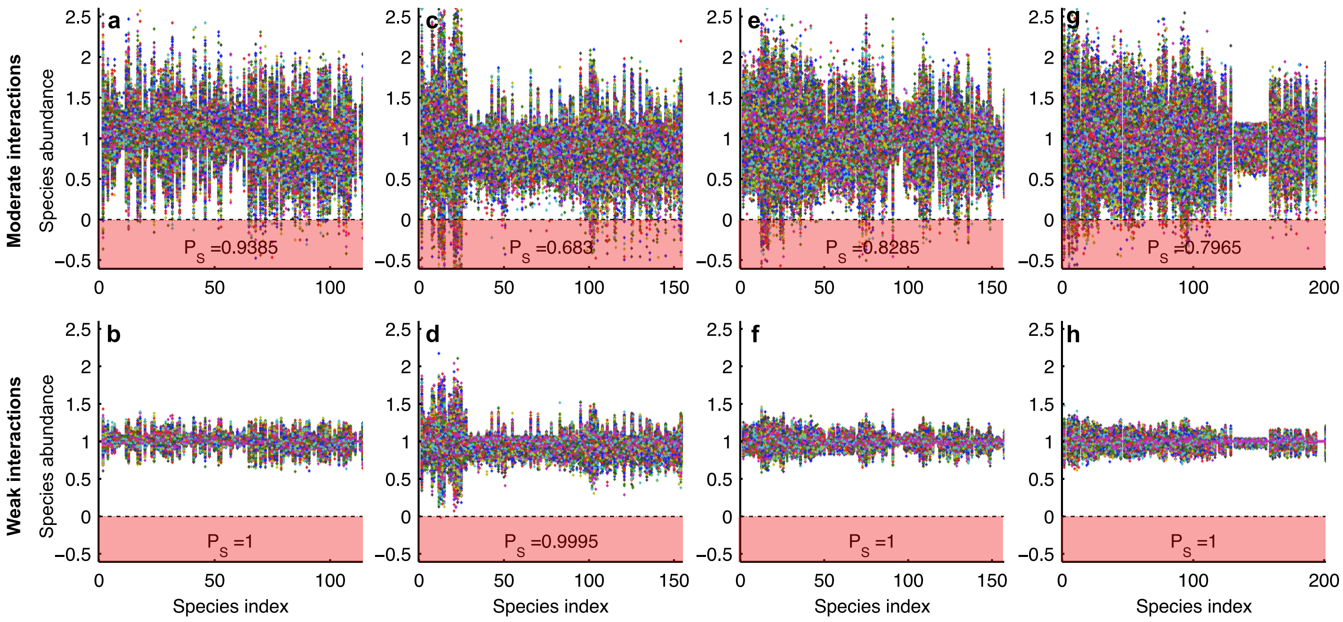

Supplementary Figure H. Species abundance at equilibrium in four empirical food webs with the mean structural vector. (**a-b**) Stony, (**c-d**) Broom, (**e-f**) El-Verde and (**g-h**) Little Rock Lake. The mean of each x_i^* is always one due to the mean structural vector, but the variance depends on the species index i, i.e. on the species role in the web. 2000 simulations of x^* are represented in each case. The mean structural vector has been simulated via Monte-Carlo methods (200 simulations).

Proof. Consider $A/\sqrt{CS}$ so that $\sigma < |\theta|$. This is equivalent to say that $B = \left(\theta I + \frac{1}{\sqrt{CS}} A\right)$ possesses only eigenvalues with negative real parts. Define now the symmetric matrix $\tilde{B} = DB + B^T D$, where $D = \text{diag}\left(\frac{1}{\sqrt{2}}\right)$. Each non-diagonal entry of the matrix $\tilde{B}$ are independent of the others up to symmetry, and have mean zero and standard deviation σ. By the Wigner semicircle law and May's criterion, its eigenvalues are almost surely all negative when $S \to \infty$. The matrix B is thus dissipative when $S \to \infty$, which implies that any feasible equilibrium of the model (S1) is locally stable (see [76]). ■

Note that feasibility is required to conclude on the stability of the equilibrium. Indeed, we illustrate in Suppl. Fig. J that a stable equilibrium in the sense of $J = (\theta I + A/\sqrt{CS})$ (as in [10], i.e. so that $\sqrt{SC}\sigma < |\theta|$), is stable in the sense of $J = \text{diag}(x^*)(\theta I + A/\sqrt{CS})$ only when it is feasible, which we showed is never the case in large systems (see Thm. S.3.3).

S.4.1.2 Weak interactions

In the case of weak interactions, May's criterion [10] and the criteria for competition and predation-prey established by Allesina and Tang [15] are asymptotically trivially satisfied if the parameters are chosen so that the (deterministic) equilibrium is admissible. Indeed, the

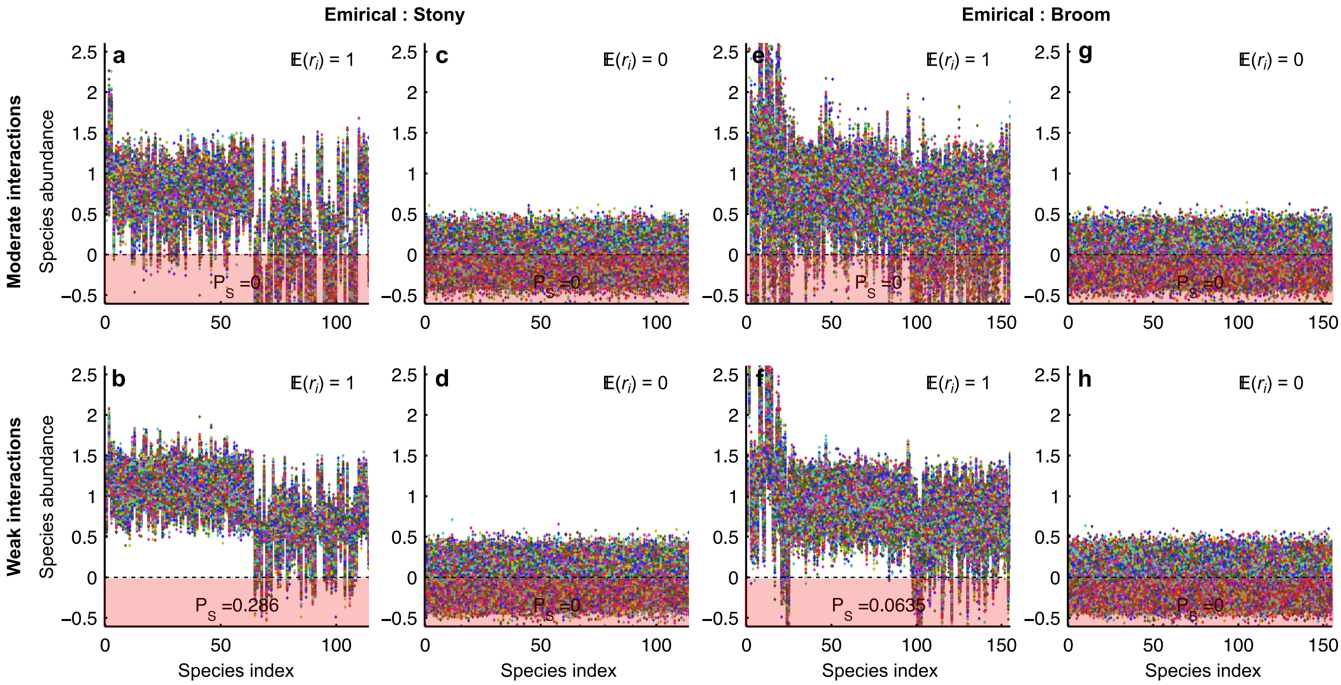

Supplementary Figure I. Species abundance at equilibrium in two empirical food webs with random growth rates. (**a-d**) Stony and (**e-h**) Broom. The growth rates are i.i.d. with mean one (**a-b** and **e-f**) or zero (**c-d** and **g-h**) and standard deviation fixed to 0.15. 2000 simulations of x^* are represented in each case.

criterion becomes $\sigma/\sqrt{SC} < |\theta|$ and $\sigma/\sqrt{SC} \to 0$ for $S \to \infty$.

Concerning mutualistic networks, the criterion in [15] is non-trivial. We show that this criterion still holds under the additional condition that x^* is feasible:

Proposition S.4.2. *Under weak interactions (Assumptions S.3.4) and for a mutualistic interaction matrix A, x^* is locally stable when x^* is feasible and when*

$$\mathbb{E}(|a_{ij}|) < |\theta|. \tag{S11}$$

Proof. the inequality (S11) has been computed in [15] using the Geršgorin circles [78]. With analogous arguments as in the proof of Prop S.4.1, we define the symmetric matrix $\tilde{B} = DB + B^T D$, where $D = \text{diag}\left(\frac{1}{\sqrt{2}}\right)$ and $B = \left(\theta I + \frac{1}{CS}A\right)$. Its largest eigenvalue has been computed in [73] and is given by $\sqrt{2}\mathbb{E}(|a_{ij}|)+\sqrt{2}\theta$, which is negative when (S11) holds. B is thus dissipative and consequently the equilibrium locally stable [76]. ∎

S.4.2 Structured models

Determining stability criteria in structured models is mathematically difficult, since the entries of the resulting random matrix $J(x^*)$ can become highly dependent. In this situation, a direct use of the classical results in [33, 79, 80] is no longer possible. In Suppl. Fig. K, we illustrate

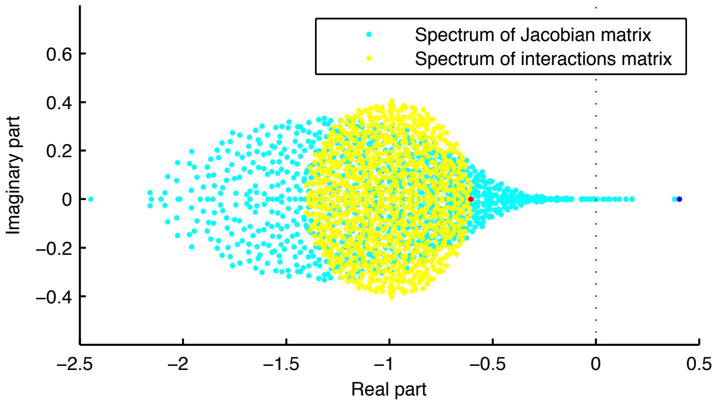

Supplementary Figure J. Spectra of the interactions matrix and the Jacobian matrix for $\delta = 0.5$ and $S = 1000$. The spectrum of the interactions matrix $(\theta I + A/\sqrt{CS})$ is represented in yellow and the eigenvalue with largest real part is highlighted in red. With this renormalisation, the convergence result of Girko [77] is recovered and, since the parameters are $\sigma = 0.4$, $C = 1$ and $\theta = -1$, May's criterion holds. However, the equilibrium is very unlikely to be feasible with $S = 1000$ (see Fig. 1, Main text), so that the criterion of Prop. S.4.1 is violated and the resulting equilibrium is not stable. This is illustrated by the spectrum of the Jacobian matrix $J(x^*)$ in cyan. The eigenvalue with largest real part is highlighted in blue.

some spectra of A and $J(x^*)$ for 50 networks of the three structured models to evidence their difference. We find that a link between feasibility and stability is very likely to exist, in a similar way as for unstructured networks.

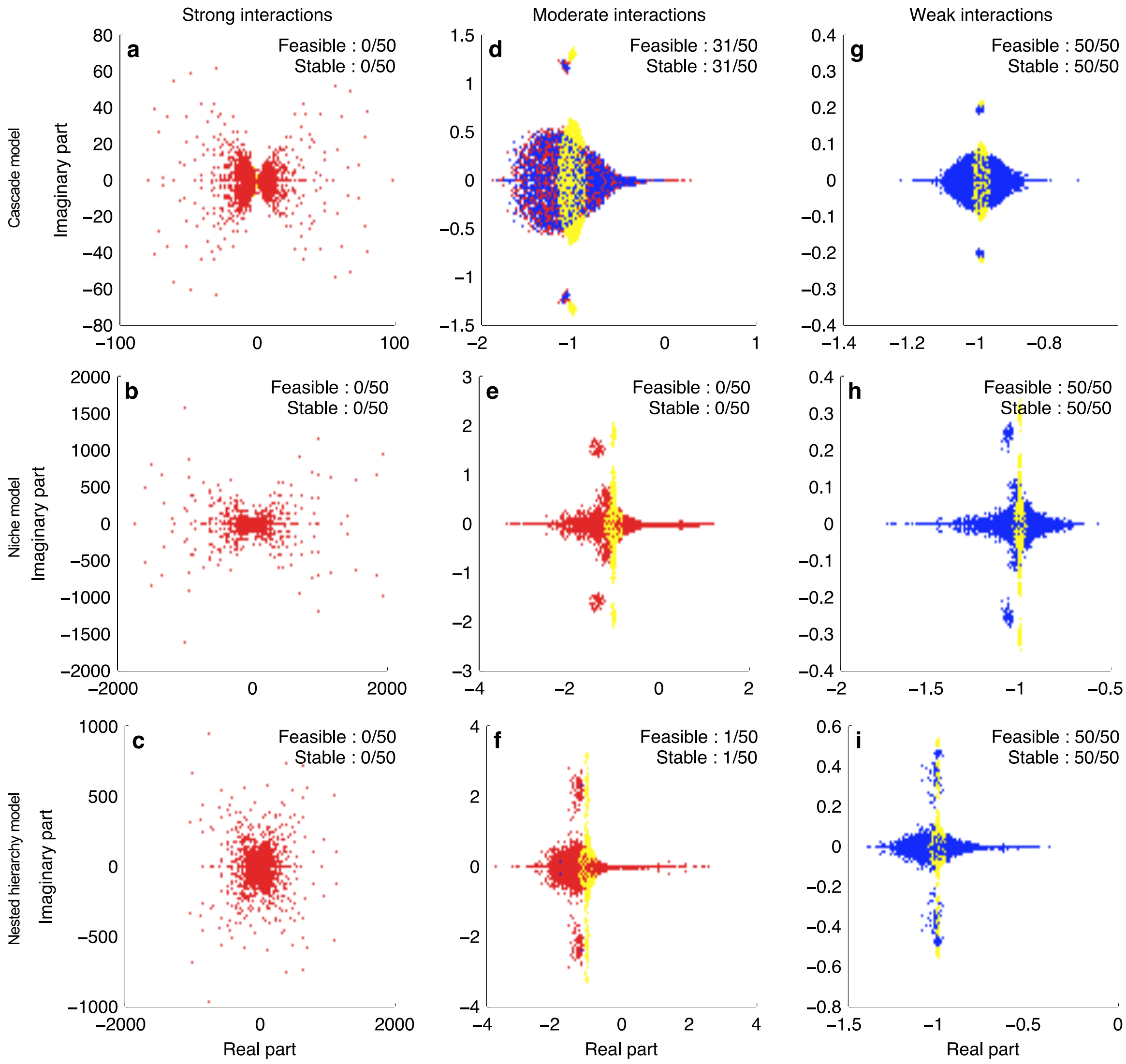

Supplementary Figure K. Spectra of the Jacobian matrix for different values of δ in structured models. The eigenvalues of $J(x^*)$ are plotted in blue when x^* is feasible, and in red otherwise; the eigenvalues of the interactions matrix $(\theta I + A/\sqrt{CS})$ is represented in yellow. (**a** - **c**) For strong interactions, the systems are degenerate for the three models, i.e. never feasible nor stable. (**d** - **i**) For moderate interactions and weak interactions, we observe the same kind of relationship between feasibility and stability as for unstructured models. For the cascade model (d, g), x^* converges to a deterministic constant; for the niche and nested-hierarchy models (e, f, h, i), x^* converges on a compact support, which can include negative values. It is thus necessary to tune the parameters to obtain feasibility; here, we chose $S = 150$, $\sigma = 0.4$, $C = 0.25$ and $\theta = -1$ in all cases.

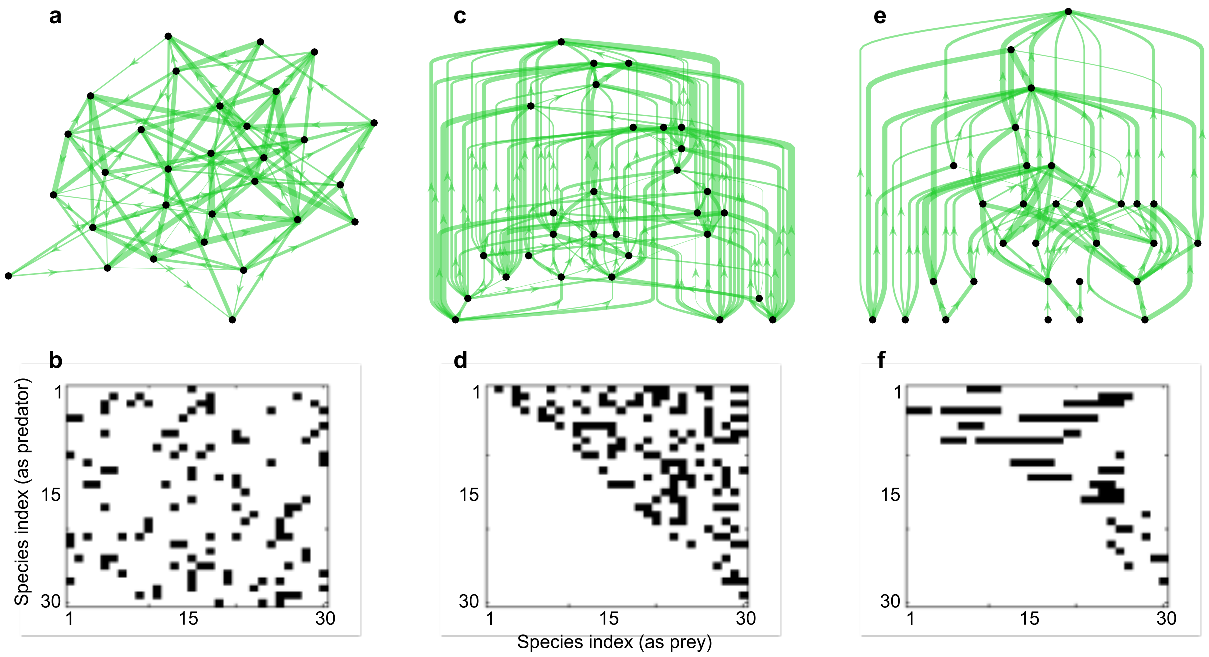

Supplementary Figure L. Illustration of some predator-prey networks and adjacency matrices. (**a-b**) Unstructured model for predation. (**c-d**) The cascade model. (**e-f**) The niche model. The parameters are $C = 0.25$ and $S = 30$. The interaction strengths are sampled at random, according to the procedure described in Section S.2.3. Thicker green arrows represent larger interaction strengths. Only positive interactions are represented.

S.5 Feasibilty with a Holling type II functional response

The question of feasibility is of course not restricted to the models studied in the present work. Instead of the Lotka-Volterra dynamics which was used in the previous and for which the interactions follows a mass action law (Holling type I [81]), we could for example explore the same question with different type of functional response.

As an illustration, we consider the following system of differential equations [82] in the predator-prey model of Section S.2.3.1,

$$\frac{\mathrm{d}x_i}{\mathrm{d}t} = x_i \left(r_i + \theta x_i + \sum_{j \in \mathrm{Prey}(i)}^{S} \frac{\frac{a_{ij}}{(CS)^\delta}}{h + \sum\limits_{k \in \mathrm{Prey}(i)} \frac{a_{ik}}{(CS)^\delta} x_k} x_j + \sum_{j \in \mathrm{Pred}(i)}^{S} \frac{\frac{a_{ij}}{(CS)^\delta}}{h + \sum\limits_{k \in \mathrm{Prey}(j)} \frac{a_{jk}}{(CS)^\delta} x_k} x_j \right) \tag{S12}$$

for $i \in \mathcal{S} = \{1, \ldots, S\}$, where $h > 0$ is some constant, Prey(i) is the set of preys of species i and Pred(i) its set of predators. This kind of functional response changes drastically the asymptotic behavior of the probability of feasibility as illustrated in Suppl. Fig. M, where we see it converging to one in the case of moderate interactions ($\delta = 1/2$).

The reason for this difference with the Holling type I models comes from the saturation term. In this mean-field framework, Holling type II dynamics acts has a buffer of interactions by taking into account the number of preys and predators in the system. Asymptotically, it makes interactions weak, whatever the value of the parameter δ. Hence, it ensures the feasibility of the equilibrium.

To understand this, consider the global effect of any species $j \in \mathcal{S}$ on i given by the generic term

$$b_{ij} = \frac{a_{ij}/(CS)^\delta}{h + \sum_{k \in \mathrm{Prey}(i)} a_{ik}/(CS)^\delta x_k} x_j \,.$$

To simplify, assume that each a_{ij} is equal to its conditional mean $a_+ \geqslant 0$, if i is a predator or $a_- \leqslant 0$ if i is a prey. Then, considering that, in this predator-prey model, the number of preys and predators of species i is a contant time CS, that is $|\mathrm{Prey}(i)| = |\mathrm{Pred}(i)| = O(CS)$ in Landau notation, b_{ij} can be approximated by

$$b_{ij} \approx \frac{a_\pm}{h \cdot (CS)^\delta + \gamma \cdot a_+ \, CS \, \bar{x}} x_j \approx \frac{\tilde{\gamma}}{CS} x_j,$$

where $a_\pm = a_+$ if j is a predator and $a_\pm = a_-$ otherwise, $\bar{x}$ is the empirical mean of all considered x_j, γ and $\tilde{\gamma}$ are some positive constant. Note that the last approximation holds for $0 \leqslant \delta \leqslant 1$ and S large. Therefore, the order of an interaction b_{ij} is $1/CS$ (weak interaction) no matter what regime δ was initially set.

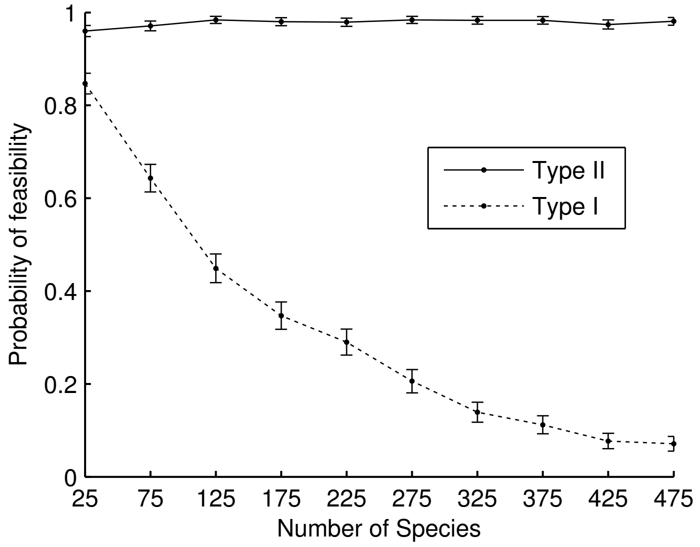

Supplementary Figure M. Probability of feasibility under moderate interactions $\delta = 1/2$ when considering Holling type II (continuous line) and Holling type I (dotted line) functional responses. The system considered is the random predator-prey model. The 95%-confidence intervals are calculated over 1000 simulations. Holling type II reacts as if the interactions were weak in a mass-action dynamics. In particular, the probability of feasibility approaches one in this case. For comparison purposes, we set $r = \mathbb{1}$, $\sigma = 0.4$, $C = 0.4$, and $\theta = -1$ in both cases and in these simulations, $h = 1$. The system at equilibrium becomes non-linear but has been solved numerically with the method fsolve in Matlab R2012b.

Supplementary references

[1] Kingsland SE. Modelling Nature: Episodes in the History of Population Ecology. The University of Chicago Press; 1985.

[2] Cuddigton K. The "Balance of Nature" Metaphor and Equilibrium in Population Ecology. Biology and Philosophy. 2001;16:463–479.

[3] MacArthur R. Fluctuations of animal populations and a measure of community stability. Ecology. 1955;36:533–536.

[4] Odum EP. Fundamentals of Ecology. Saunders Co Philadelphia; 1953.

[5] Elton CS. The Ecology of Invasion by Animals and Plants. Menthuen, London; 1958.

[6] Levins R. Evolution in Changing Environments. Princeton University Press; 1968.

[7] Levins R. The Qualitative Analysis of Partailly Specified Systems. Annals of the New York Academy of Sciences. 1974;231:123–138.

[8] Gardner M, Ashby W. Connectance of Large Dynamic (Cybernetic) Systems: Critical Values for Stability. Nature. 1970;228:784.

[9] May RM. Stability in multispecies community models. Mathematical Biosciences. 1971;12:59–79.

[10] May RM. Will a large complex system be stable? Nature. 1972;238:413–414.

[11] May RM. Stability and complexity in model ecosystems. Princeton University Press; 1973.

[12] Wigner EP. On the distribution of the roots of certain symmetric matrices. Annals of Mathematics. 1958; p. 325–327.

[13] Ginibre J. Statistical ensembles of complex, quaternion, and real matrices. Journal of Mathematical Physics. 1965;6(3):440–449.

[14] Bersier LF. A History of the Study of Ecological Networks. In: Kepes F, editor. Biological Networks. vol. 3. New Jersey: World Scientific Pub Co Inc; 2007. p. 365–421.

[15] Allesina S, Tang S. Stability Criteria for Complex Ecosystems. Nature. 2012;483:205–208.

[16] Allesina S, Grilli J, Barabás G, Tang S, Aljadeff J, Maritan A. Predicting the stability of large structured food webs. Nature communications. 2015;6.

[17] Cohen JE, Briand F, Newman CM. Community food webs. vol. 20 of Biomathematics. Springer-Verlag; 1990.

[18] Williams RJ, Martinez ND. Simple rules yield complex food webs. Nature. 2000;404(6774):180–183.

[19] Gellner G, McCann K. Consistent role of weak and strong interactions in high-and-low-diversity trophic food webs. Nature communications. 2016;7:11180.

[20] James A, Plank M, Rossberg A, Beecham J, Emmerson M, Pitchford J. Constructing Random Matrices to Represent Real Ecosystems. The American Naturalist. 2015;185(5):680–692.

[21] Johnson S, Dominguez-Garcia V, Donetti L, Munoz M. Trophic coherence determines food-web stability. PNAS. 2014;16(17923–17928).

[22] Roberts A. The stability of a feasible random ecosystem. Nature. 1974;251:607–608.

[23] Logofet DO. Matrices and graphs: stability problems in mathematical ecology. CRC Press; 1993.

[24] Bastolla U, Lassig M, Manrubia S, Valleriani A. Biodiversity in model ecosystems, I: coexistence conditions for competing species. Journal of Theoretical Biology. 2005;235(4):521–530.

[25] Nattrass S, Baigent S, Murrell DJ. Quantifying the Likelihood of Co-existence for Communities with Asymmetric Competition. Bulletin of Mathematical Biology. 2012;74:2315–2338.

[26] Rohr R, Saaveda S, Bascompte J. On the structural stability of mutualistic systems. Science. 2014;345(6195).

[27] Vandermeer JH. The community matrix and the number of species in a community. The American Naturalist. 1970;104(935):73–83.

[28] Cattin MF, Bersier LF, Banašek-Richter C, Baltensperger R, Gabriel JP. Phylogenetic constraints and adaptation explain food-web structure. Nature. 2004;427(6977):835–839.

[29] Gillman M, Hails R. An Introduction to Ecological Modelling. Putting Practice into Theory. Blackwell Science, Oxford; 1997.

[30] McCann K, Hastings A, Huxel G. Weak trophic interactions and the balance of nature. Nature. 1998;395:794–798.

[31] Emmerson M, Yearsley J. Weak interactions, omnivory and emergent food-web properties. Proceedings of the Royal society of London Series B Biological sciences. 2004;271:397–405.

[32] Stouffer DBB, Camacho J, Guimera R, Ng CA, Nunes Amaral LA. Quantitative patterns in the structure of model and empirical food webs. Ecology. 2005;86(5):1301–1311.

[33] Geman S, Hwang C. A Chaos Hypothesis for Some Large Systems of Random Equations. Zeitschrift für Wahrscheinlichkeitstheorie und Verwandte Gebiete. 1982;60:291–314.

[34] De Angelis DL. Stability and Connectance in Food Web Models. Ecology. 1975;56:238–243.

[35] Dunne JA, Williams RJ, Martinez ND. Network structure and biodiversity loss in food webs: robustness increases with connectance. Ecology letters. 2002;5(4):558–567.

[36] Okuyama T, Holland JN. Network structural properties mediate the stability of mutualistic communities. Ecology Letters. 2008;11(3):208–216.

[37] Thébault E, Fontaine C. Stability of ecological communities and the architecture of mutualistic and trophic networks. Science. 2010;329(5993):853–856.

[38] O'Gorman E, Emmerson M. Manipulating interaction strengths and the consequence for trivariate patterns in a marine food web. Advances in Ecological Research. 2010;42:301–419.

[39] Twomey M, Jacob U, Emmerson M. Perturbing a marine food web: consequences for food web structure and trivariate terms. Advances in Ecological Research. 2012;47:349–409.

[40] Rooney N, McCann K, Gellner G, Moore JC. Structural asymmetry and the stability of diverse food webs. Nature. 2006;442(20):265–269.

[41] Holt RD, Hoopes MF. Food web dynamics in a metacommunity context: modules and beyond. In: Holyoak M, Leibold MA, Holt RD, editors. Metacommunities: spatial dynamics and ecological communities. University of Chicago Press; 2005.

[42] Banašek-Richter C, Bersier LF, Cattin MF, Baltensperger R, Gabriel JP, Merz Y, et al. Complexity in quantitative food webs. Ecology. 2009;90(6):1470–1477.

[43] Drossel B, McKane A, Quince C. The impact of non-linear functional responses on the long-term evolution of food web structures. Journal of Theoretical Biology. 2004;229:539–548.

[44] Allesina S, Alonso D, Pascual M. A General Model for Food Web Structure. Science. 2008;320:658–661.

[45] Rossberg AG, Ishii R, Amemiya T, Itoh K. The top-down mechanism for body-mass-abundance scaling. Ecology. 2008;89(2):567–580.

[46] Laska M, Wootton J. Theoretical concepts and empirical approaches to measuring interaction strength. Ecology. 1998;79(2):461–476.

[47] Berlow E, Neutel A, Cohen J, de Ruiter P, Ebenman B, Emmerson Mea. Interaction strengths in food webs: issues and opportunities. Journal of Animal Ecology. 2004;73(3):585–598.

[48] Arditi R, Bersier LF, Rohr RP. The perfect mixing paradox and the logistic equation: Verhulst vs. Lotka. Ecosphere. In press;.

[49] Neutel AM, Thorne M. Interaction strengths in balanced carbon cycles and the absence of a relation between ecosystem complexity and stability. Ecology Letters. 2014;17(6):651–661.

[50] Jacquet C, Moritz C, Morissette L, Legagneux P, Massol F, Archambault P, et al. No complexity-stability relationship in natural communities. Nature communications. 2016;7:12573.

[51] Emmerson M, Raffaelli D. Predator-prey body size, interaction strengths and the stability of a real food web. Journal of Animal Ecology. 2004;73(3):399–409.

[52] Moore J, de Ruiter P, Hunt H, Coleman D, Freckman D. Microcosms and soil ecology: critical linkages between fields studies and modelling food webs. Ecology. 1996;77(3):694–705.

[53] Christensen V, Pauly D. Ecopath II-a software for balancing steady-state ecosystem models and calculating network characteristics. Ecological Modelling. 1992;61(3–4):169–185.

[54] Neutel AM, Heesterbeek JAP, van de Koppel J, Hoenderboom G, Vos A, Kaldeway C, et al. Reconciling complexity with stability in naturally assembling food webs. Nature. 2007;449(7162):599–602.

[55] Brose U, Williams RJ, Martinez ND. Allometric scaling enhances stability in complex food webs. Ecology Letters. 2006;9(11):1228–1236.

[56] Arditi R, Ginzburg LR. How Species Interact: Altering the Standard View on Trophic Ecology. Oxford University Press; 2012.

[57] Abrams PA. Why ratio dependence is (still) a bad model of predation. Biological Reviews. 2015;90(3).

[58] Kondoh M. Foraging Adaptation and the Relationship Between Food-Web Complexity and Stability. Science. 2003;299(5611):1388–1391.

[59] Gross T, Ebenhöh W, Feudel U. Enrichment of foodchain stability: the impact of different forms of predator-prey interaction. Journal of Theoretical Biology. 2004;227(3):349–358.

[60] Gross T, Feudel U. Generalized models as a universal approach to the analysis of nonlinear dynamical systems. Physical Review E. 2006;73(1):016205.

[61] Gross T, Rudolf L, Levin S, Dieckmann U. Generalized Models Reveal Stabilizing Factors in Food Webs. Science. 2009;325(5941):747–750.

[62] Jones CG, Lawton JW, Shachak M. Organisms as ecosystem engineers. Oikos. 1994;69:373–386.

[63] Odling Smee J, Laland K, Feldman M. Niche Construction: The Neglected Process in Evolution. Princeton University Press; 2003.

[64] Arditi R, Michalski J, Hirzel A. Rheagogies: Modelling non-trophic effects in food webs. Ecological Complexity. 2005;2(3):249–258.

[65] Fontaine C, Guimarães J P R, Kéfi S, Loeuille N, Memmott J, van der Putten WH, et al. The ecological and evolutionary implications of merging different types of networks. Ecology Letters. 2011;14(11):1170–1181.

[66] Pocock MJO, Evans DM, Memmott J. The Robustness and Restoration of a Network of Ecological Networks. Science. 2012;335(6071):973–977.

[67] Tabor M. Chaos and Integrability in Nonlinear Dynamics: An Introduction. Wiley; 1989.

[68] Logofet DO. Stronger-than-Lyapunov notions of matrix stability, or how "flowers" help solve problems in mathematical ecology. Linear Algebra Appl. 2005;398:75–100.

[69] Lotka AJ. Elements of Physical Biology. Williams and Wilkins Company; 1925.

[70] Volterra V. Variazioni e fluttuazioni del numero d'individui in specie animali conviventi. Memoria della Regia Accademia Nazionale dei Lincei. 1926;6:31–113.

[71] Rossberg AG. Food Webs and Biodiversity. Wiley-Blackwell; 2013.

[72] Geman S. A limit theorem for the norm of random matrices. Annals of Probability. 1980;8:252–261.

[73] Füredi Z, Komlós J. The eigenvalues of random symmetric matrices. Combinatorica. 1981;1(3):233–241.

[74] Hofmann-Credner K, Stolz M. Wigner theorems for random matrices with dependent entries: Ensembles associated to symmetric spaces and sample covariance matrices. Electronic Communications in Probability. 2008;13:401–414.

[75] Schmid-Araya JM, Schmid PE, Robertson A, Winterbottom J, Gjerlov C, Hildrew AG. Connectance in stream food webs. Journal of Animal Ecology. 2002;71:1056–1062.

[76] Svirezhev YM, Logofet DO. Stability of Biological Communities. Mir Publishers; 1982.

[77] Girko VL. Circular law. Theory of Probability and its Applications. 1985;29(4):694–706.

[78] Geršgorin S. Über die Abgrenzung der Eigenwerte einer Matrix. Bulletin de l'Académie des Sciences de l'URSS Classe des sciences mathématiques et na. 1931;6:749–754.

[79] Tao T, Vu V, Krishnapur M, et al. Random matrices: universality of ESDs and the circular law. The Annals of Probability. 2010;38(5):2023–2065.

[80] Sommers HJ, Crisanti A, Sompolinsky H, Stein Y. Spectrum of large random asymetric matrices. Physical Review Letters. 1988;60:1895–1898.

[81] Holling CS. The Components of Predation as Revealed by a Study of Small-Mammal Predation of the European Pine Sawfly. The Canadian Entomologist. 1959;91(5):293–320. doi:10.4039/Ent91293-5.

[82] Koen-Alonso M, Yodzis P. Dealing with model uncertainty in trophodynamic models: A patagonian example. In: Ruiter Pd, Wolters V, Moore JC, editors. Dynamic food webs: multispecies assemblages, ecosystem development and environmental change. vol. 3 of Theoretical Ecology Series. Academic Press; 2005. p. 381 – 394.
